# Supplementary material for: Tunable stochastic memristors for energy-efficient encryption and computing
Source: Nat Commun. 2024 Apr 15;15:3245. doi: 10.1038/s41467-024-47488-x (PMC11018740; doi:10.1038/s41467-024-47488-x)
Supplement: Supplementary file 1 — Supplementary Information [file 41467_2024_47488_MOESM1_ESM.pdf]

SUPPLEMENTARY INFORMATION

Tunable Stochastic Memristors for Energy-Efficient Encryption and Computing

Kyung Seok Woo[1,2,3,5], Janguk Han[1,5], Su-in Yi[3], Luke Thomas[4], Hyungjun Park[1], Suhas Kumar[2,*], Cheol Seong Hwang[1,*]

[1]*Department of Materials Science and Engineering and Inter-University Semiconductor Research Center, Seoul National University, Gwanak-ro 1, Daehag-dong, Gwanak-gu, Seoul, Republic of Korea.*

[2]*Sandia National Laboratories, Livermore, CA, USA*

[3]*Department of Electrical and Computer Engineering, Texas A&M University, College Station, TX, USA*

[4]*Applied Materials Inc., Santa Clara, CA, USA*

[5]*These two authors contributed equally.*

[*]*Correspondence: S.K. (email: su1@alumni.stanford.edu), C.S.H. (e-mail: cheolsh@snu.ac.kr)*

Table of contents

1. **Metal-filament-based memristor**
2. **Scalability of the crossbar array**
3. **Karnaugh map**
4. **1-bit binary full adder/subtractor**
5. **The hardware setup for a fully encrypted memory-PUF implementation**
6. **Encrypted inference**
7. **Homomorphic encryption and federated learning**
8. **Energy and latency comparisons of memristor-based encrypted inference with traditional computing systems**

Supplementary Note 1. Metal-filament-based memristor.

1.1. Switching behavior at different Cu_xTe_{1-x} compositions.

Depending on the Cu_xTe_{1-x} composition, the memristor can be either volatile or nonvolatile[1]. The volatile characteristics observed at the low composition of Cu are attributed to the formation of minuscule Cu conductive filaments (CFs). In these CFs, the Cu atoms diffuse back to the active electrode upon voltage removal due to the high interface energy of the Cu CF (Supplementary Fig. 1a). On the other hand, higher values of x lead to the formation of stronger Cu CFs, which prevent dissolution and exhibit nonvolatile characteristics (Supplementary Fig. 1b). Cu preferentially contributes to the filament formation over Te since the electron affinity of Cu (118.4 kJ mol^{-1}) is lower than that of Te (190.2 kJ mol^{-1}).

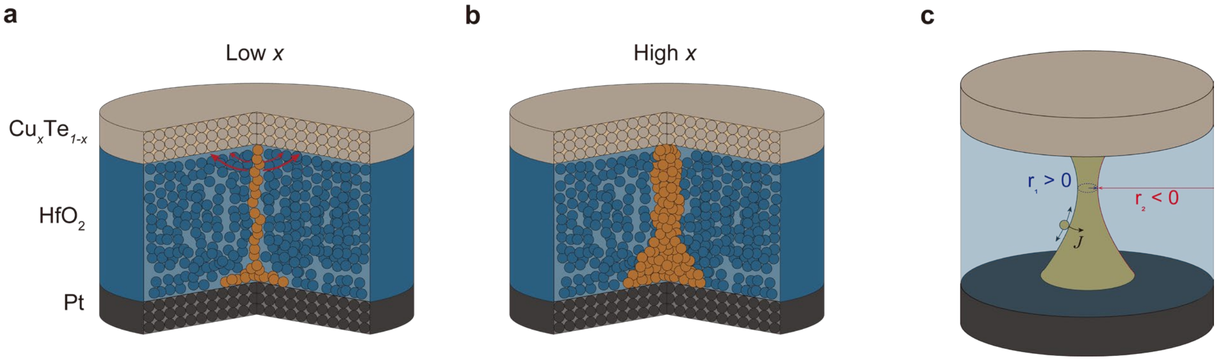

Supplementary Fig. 1 | Switching behavior of the Cu_xTe_{1-x} memristor at a, low *x* and **b,** high *x*. **c,** Cu filament formation with two radii of surface curvature.

1.2. Surface diffusion dynamics.

The primary migration mechanism is surface diffusion regarding the small size of device[2]. The dissolution time of Cu CF can be explained through a kinetic process that involves surface diffusion dynamics based on the thermodynamics Gibbs-Thomson effect. The increase in chemical potential of an atom that is transferred from a point of zero curvature to κ curvature is given by[3]

$$\mu(\kappa) = \kappa\gamma\Omega \quad \text{(S1)}$$

, where κ is the surface curvature of the filament, γ is the surface energy per unit area, and Ω is the molecular volume. Gradients of chemical potential is the function of surface curvature, and such gradients create a drift of atoms with the average velocity V defined by the Nernst-Einstein relation

$$V = -\frac{D_s}{kT}\nabla_s\,\mu(\kappa) = -\frac{D_s\gamma\Omega}{kT}\nabla_s\kappa \tag{S2}$$

, where D_s is the diffusion coefficient, k is the Boltzmann's constant, and T is the temperature. Subsequently, the surface atomic flux can be obtained by the Fick's 1st law,

$$\mathrm{J} = -\left(\frac{D_s\gamma\Omega n}{kT}\right)\nabla_s\kappa \tag{S3}$$

, where n is the number of atoms per unit area. The surface curvature κ is given by $\kappa = \frac{1}{r_1} + \frac{1}{r_2}$, where r_1 and r_2 are two surface curvature radii of the filament (Supplementary Fig. 1c). Note that the surface curvature controls the stability of the filament. When the filament diameter is smaller at low x, corresponding to a larger surface curvature, the atomic flux of the dissolution process becomes larger, leading to volatile switching. This model agrees with the experimental data concerning the coexistence of volatile and nonvolatile switching behaviors at different compliance currents, which also control the size of the filament[13].

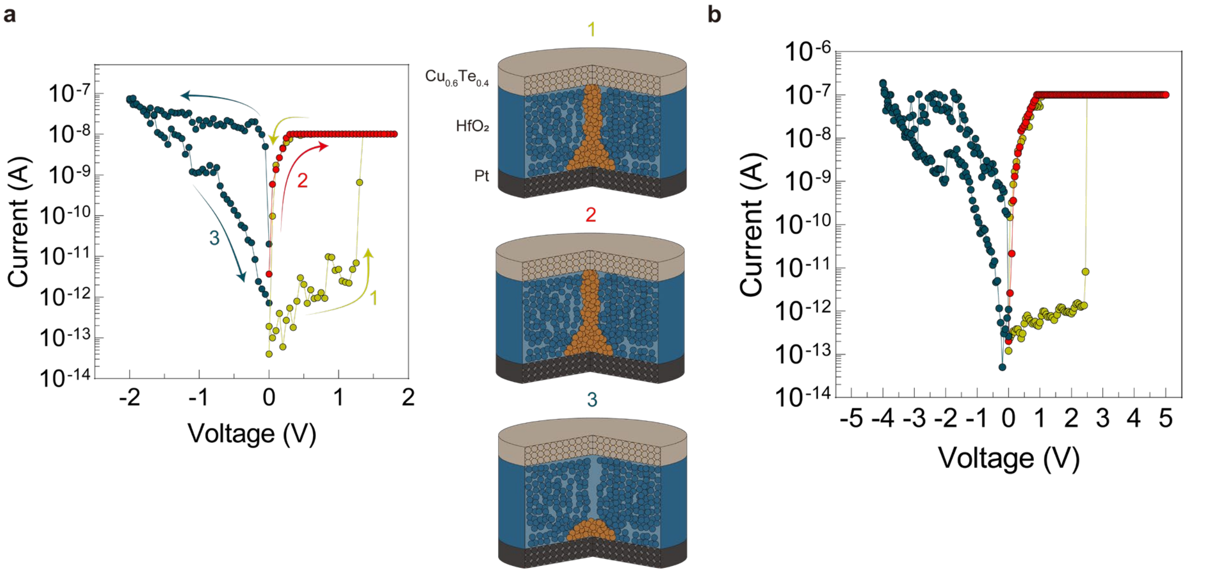

Supplementary Fig. 2 | a, Switching mechanism of the $Cu_{0.6}Te_{0.4}$ device. The *I-V* curves are reproduced from Fig. 1b. Stable Cu CFs, which are not maintained at zero bias, are formed. Therefore, the rectifying behavior is lost in the negative bias region. **b,** *I-V* curves of the $Cu_{0.3}Te_{0.7}$ device at I_{cc} = 100 nA. Similar to the $Cu_{0.6}Te_{0.4}$ device, the rectifying behavior is lost due to the stronger filament formed by higher I_{cc}. In this case, a higher reset voltage (> 4V), discussed later, was required to turn off the device completely.

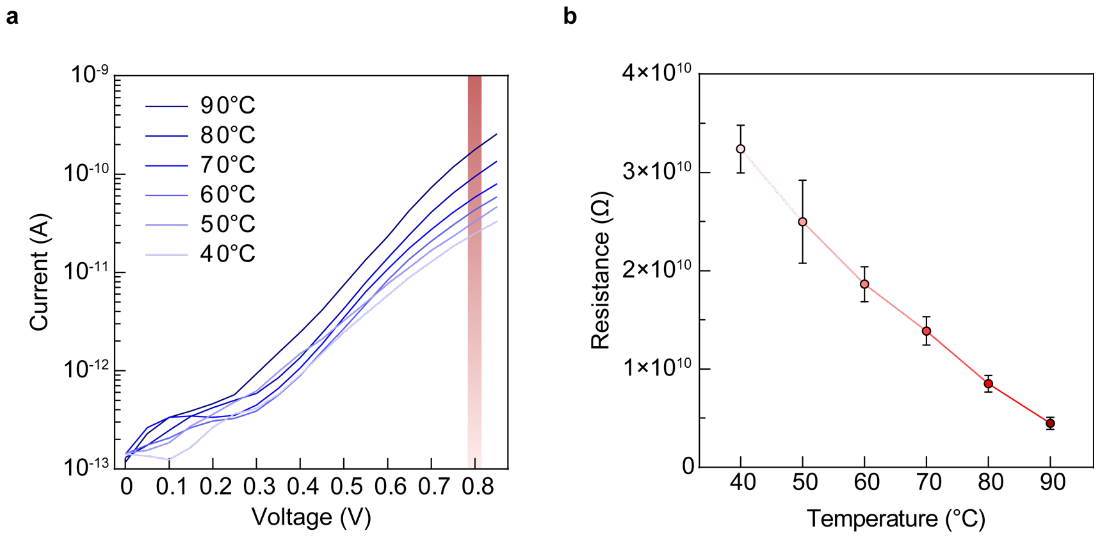

Supplementary Fig. 3 | a, *I-V* curves of the LRS device with varying temperatures. **b,** Resistance of the LRS device at 0.8 V.

Supplementary Note 2. Scalability of the crossbar array.

2.1. Sneak current issue depending on the size of the array.

A CuTeHO single device was modeled, as shown in Supplementary Fig. 4a, to evaluate the possible negative impact of sneak current on the accuracy of current reading when crossbar array size increased. Supplementary Fig. 4b quantitatively demonstrates the rectifying ratio of the CuTeHO device. For the worst-case scenario to maximize the influence of sneak current, only the (N, N) component was settled to HRS, while the remaining components were settled to LRS (N represents the number of wires in one dimension, Supplementary Fig. 4c). Supplementary Fig. 4d shows the variation in the current passed through the HRS device at (N, N) position as N increased from 2 to 1000, under the given circumstance. According to the simulation results, there was no significant change in the HRS output current up to N of 400 due to the excellent rectifying characteristic of the device. The inset of Supplementary Fig. 4d shows that the sneak current necessarily includes a path from the bottom electrode to the top electrode of the device, which the rectifying property of the CuTeHO device could suppress. However, when N exceeded 400, the worst-case HRS output current exceeded the LRS output current, leading to misinterpretation of the device's state. However, the N could be increased to 1000 when half the devices were at HRS.

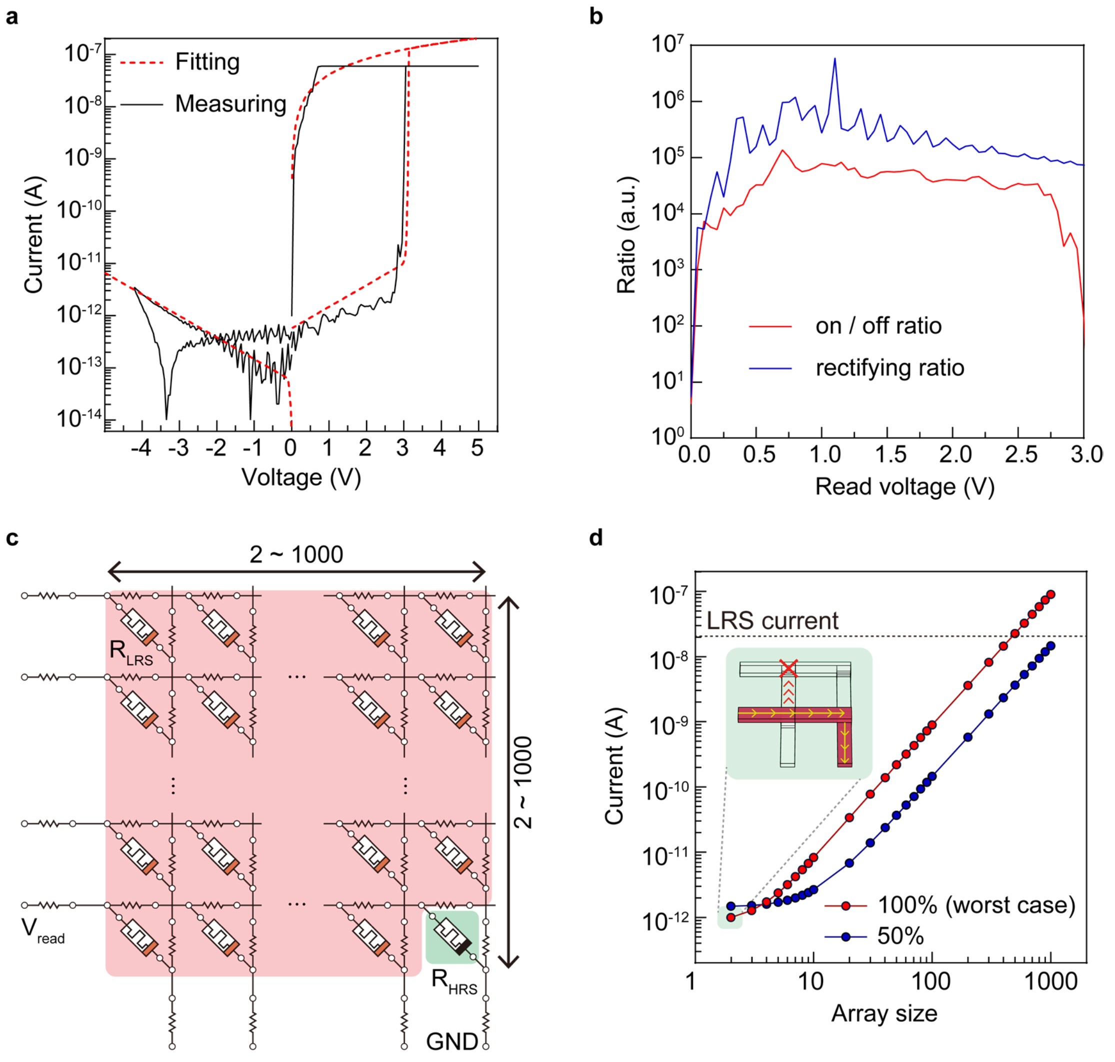

Supplementary Fig. 4 | a, *I-V* curves of the device with the fitting results (red dashed line). **b,** On/off and rectifying ratios with varying read voltages. **c,** Schematic diagram of the crossbar array. Red colors are LRS devices. **d,** The current passing through the HRS device at (*N*, *N*) position.

2.2. Impact of line resistance on the crossbar array.

The influence of line resistance was also analyzed. Supplementary Fig. 5a represents the crossbar array with the line resistance when *N* is 100. To maximize the impact of line resistance (green area), only the CuTeHO component (red area), the most influenced cell by the line resistance, was set as LRS and read. Supplementary Fig. 5b shows that the total resistance is affected only when the line resistance becomes unrealistically large because even the LRS resistance of the CuTeHO device is much higher than the line resistance.

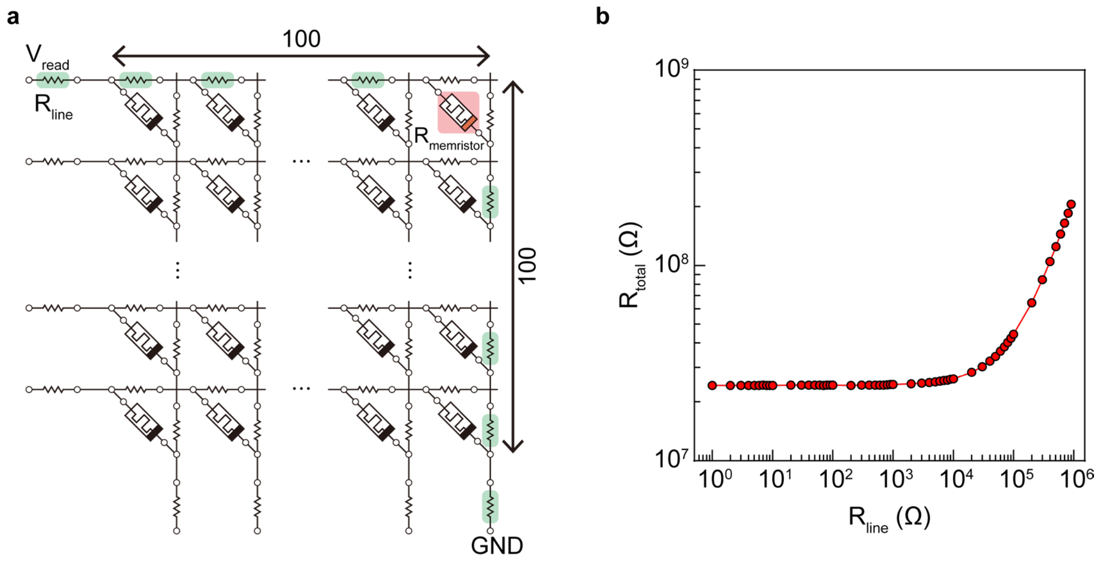

Supplementary Fig. 5 | a, Schematic diagram of the crossbar array at $N = 100$. **b,** R_{total} at different line resistance values.

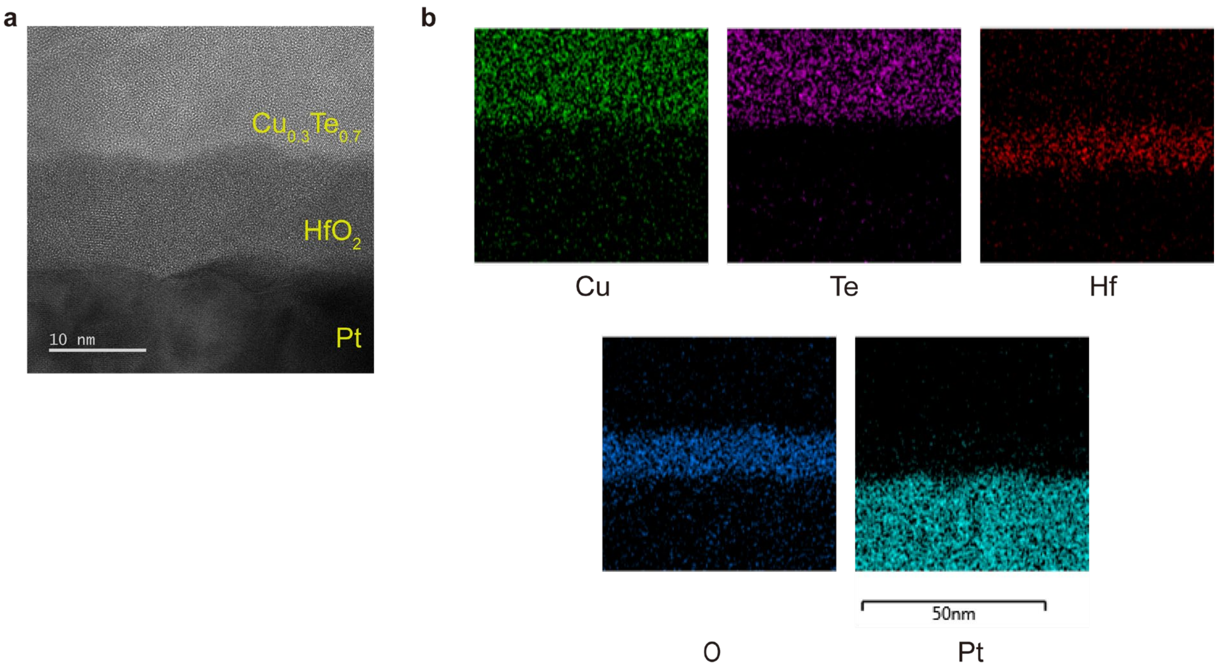

Supplementary Fig. 6 | a, Cross-sectional transmission electron microscopy (TEM) image. **b,** Energy-dispersive X-ray spectroscopy (EDS) mapping results.

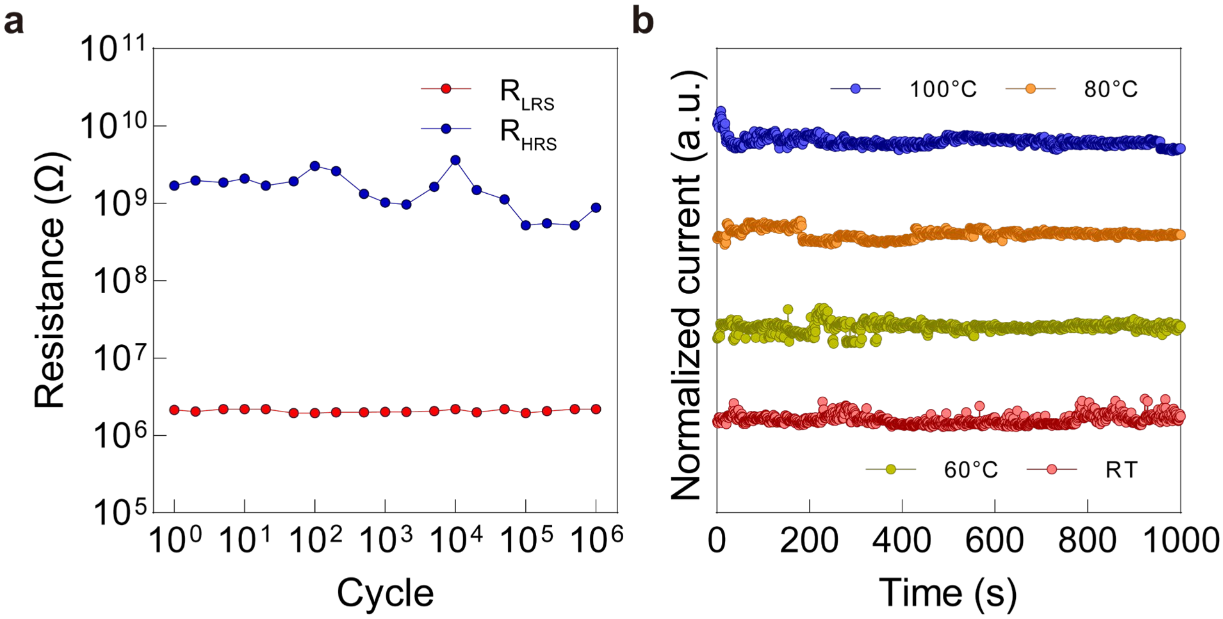

Supplementary Fig. 7 | Reliability test. a, Cycling endurance, and **b,** Retention of the $Cu_{0.3}Te_{0.7}$ device.

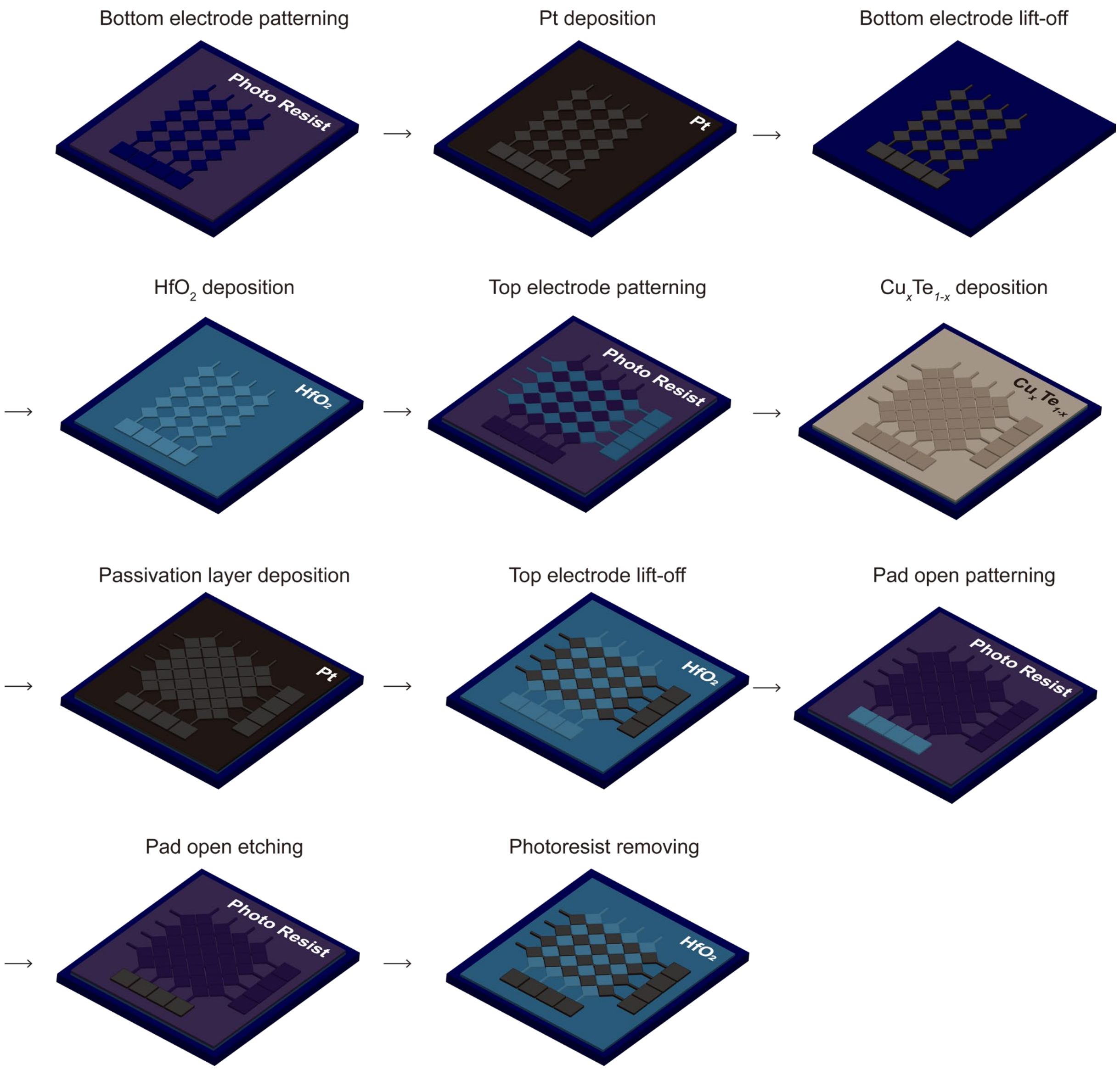

Supplementary Fig. 8 | Process flow of the memristive crossbar.

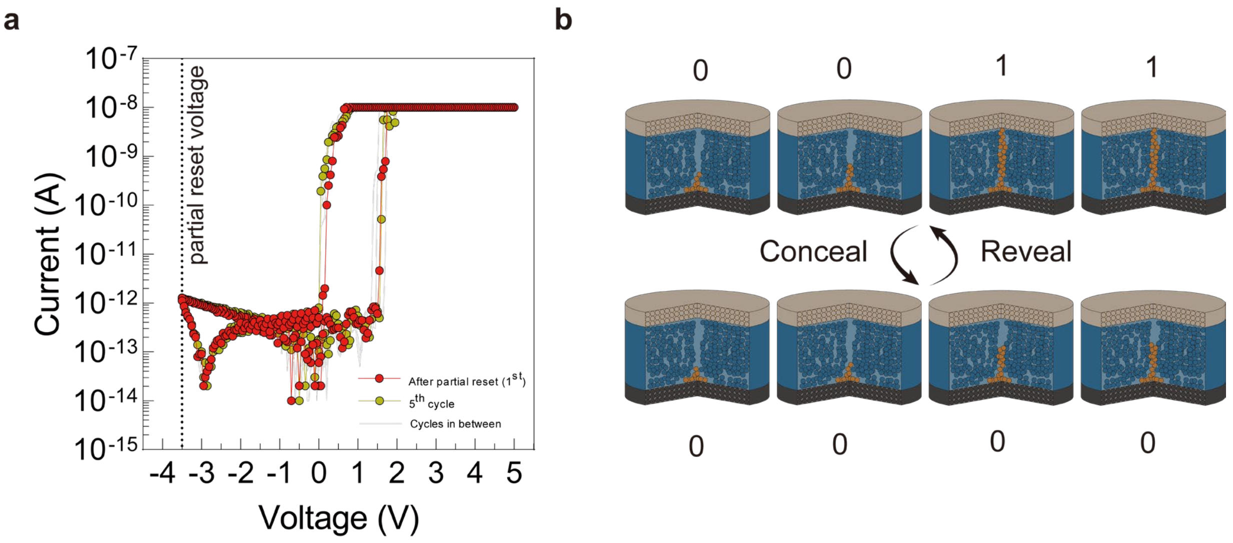

Supplementary Fig. 9 | a, *I*-*V* curves of the partial RESET. **b,** Schematic illustrations of concealing and revealing processes. With the partial RESET, memristors that are in LRS will return to HRS. Here, the filament rupture minimally occurs, allowing the device to switch to LRS at low voltage.

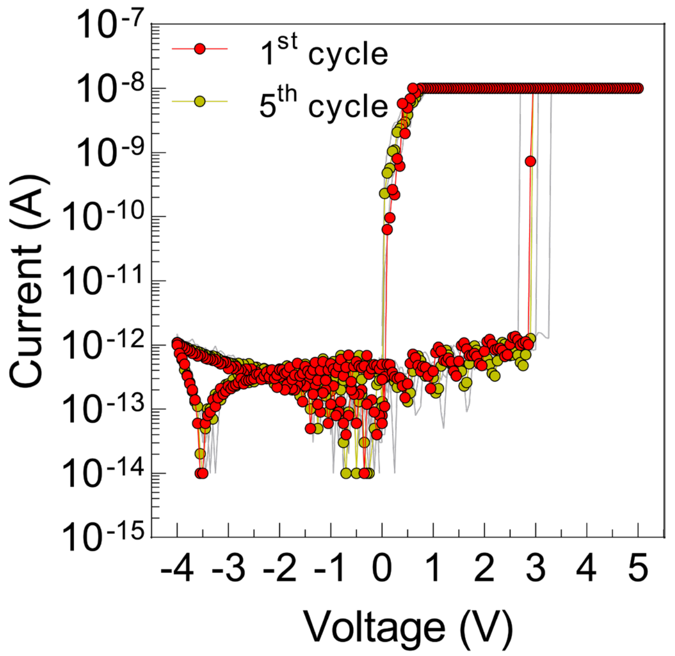

Supplementary Fig. 10 | Cycle-to-cycle variation of the CuTeHO device when the RESET voltage is applied.

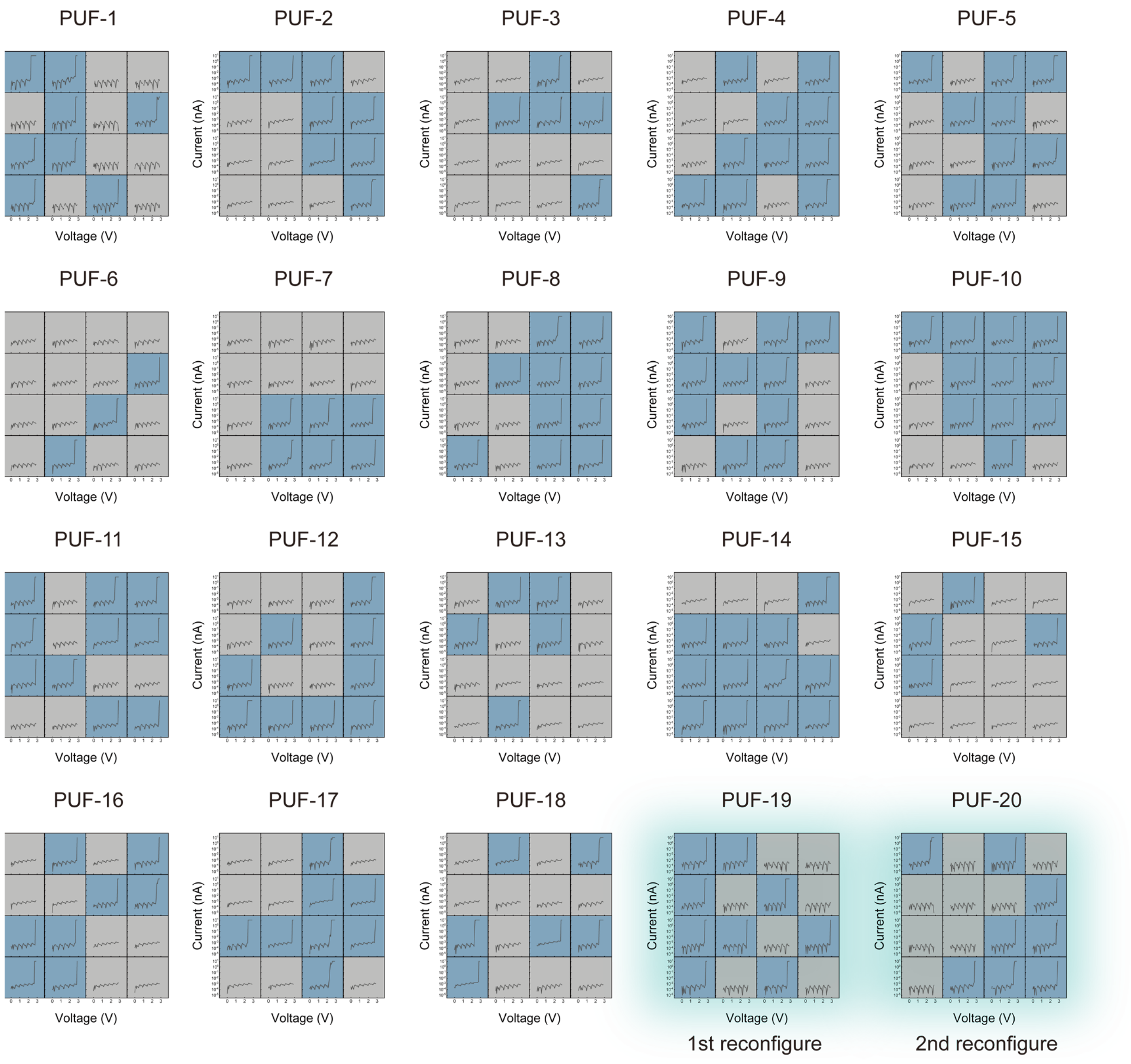

Supplementary Fig. 11 | 20 different memristive PUFs. PUF-19 and PUF-20 are reconfigured from PUF-1.

Supplementary Table 1. NIST randomness test results from the bits collected from Fig. 2a and Supplementary Fig. 11.

**Test**	**P-value**	**Pass/fail**
1. Frequency Test	0.324365	PASS
2. Frequency Test within a Block	0.666302	PASS
3. Runs test	0.970591	PASS
4. Test for the Longest Run of Ones in a Block	0.427274	PASS
5. Approximate Entropy Test	0.596730	PASS
6. Cumulative sums test	0.206239<br>0.770688	PASS

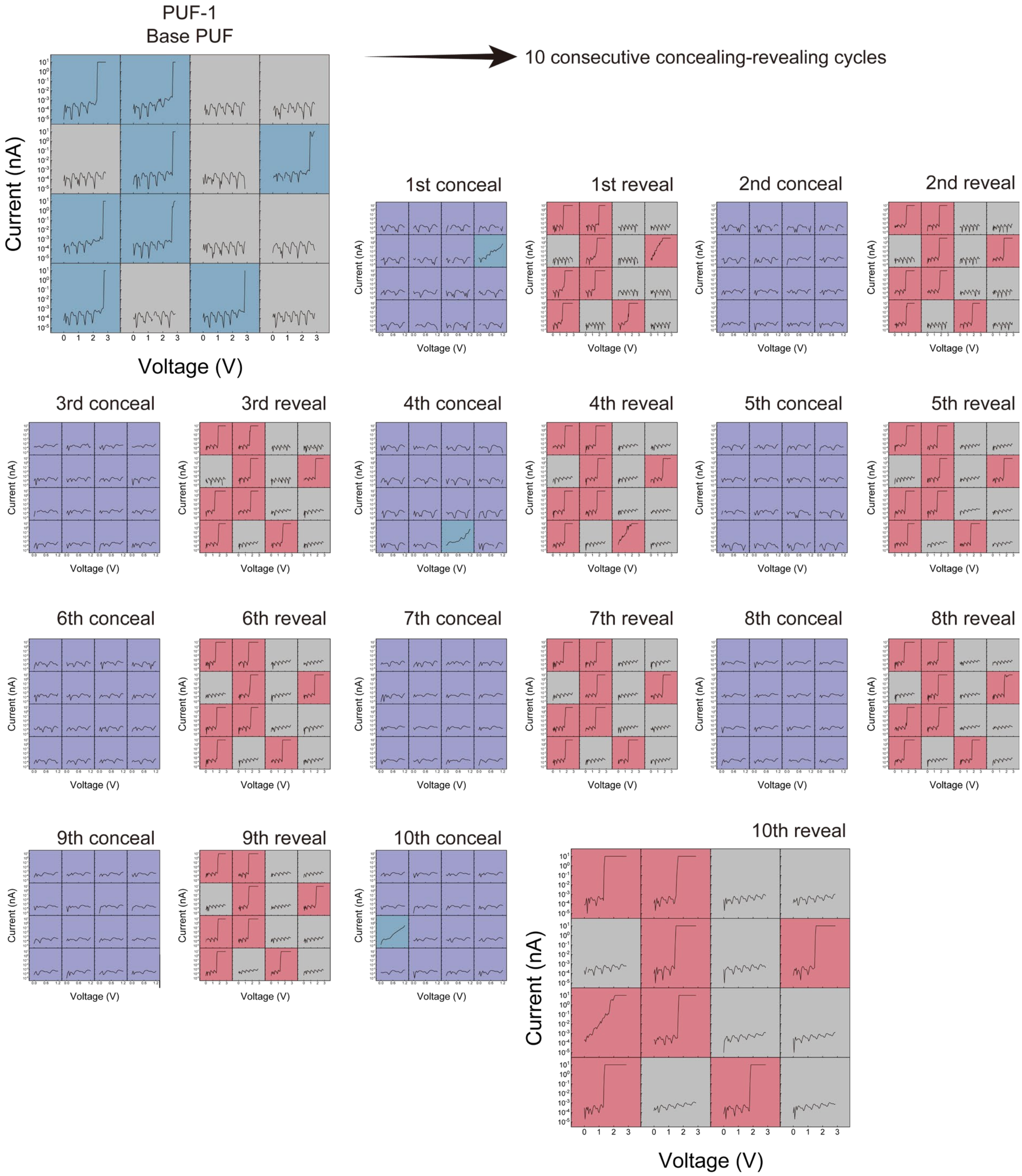

Supplementary Fig. 12 | Experimental demonstration of 10 concealing-revealing cycles.

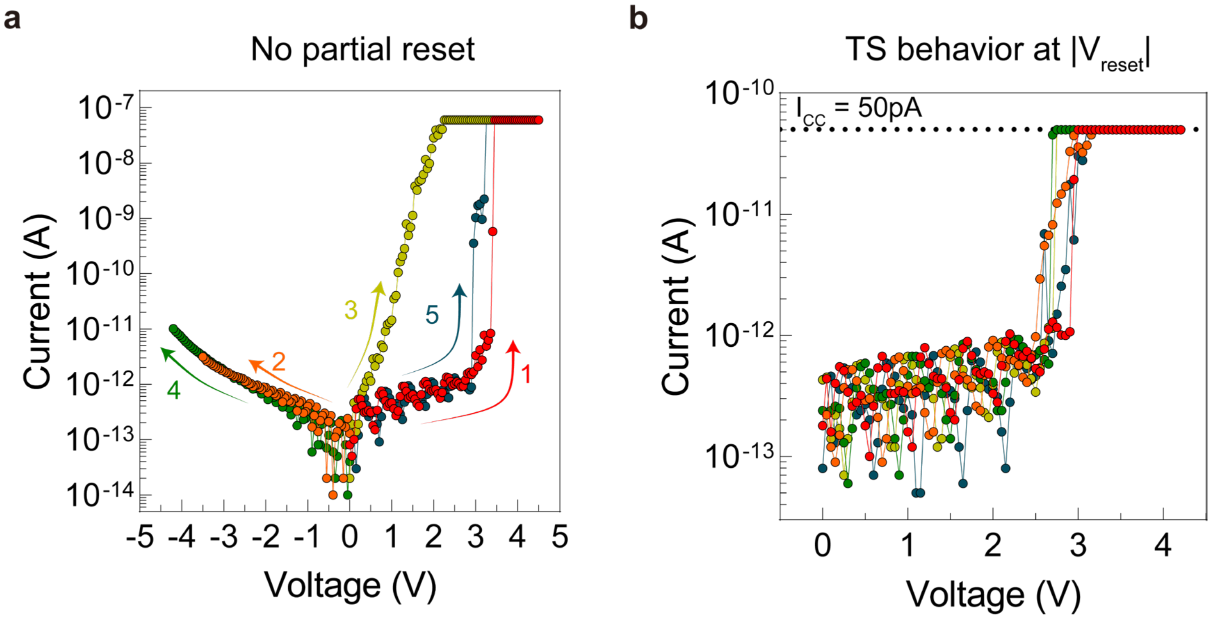

Supplementary Fig. 13 | a, *I-V* curves at different RESET voltages. The rectifying behavior is maintained at I_{cc} = 60 nA, whereas higher I_{cc} (100 nA) eliminates it due to significant filament formation, as shown in Fig. S1b. **b,** 5 consecutive cycles of V_{reset}.

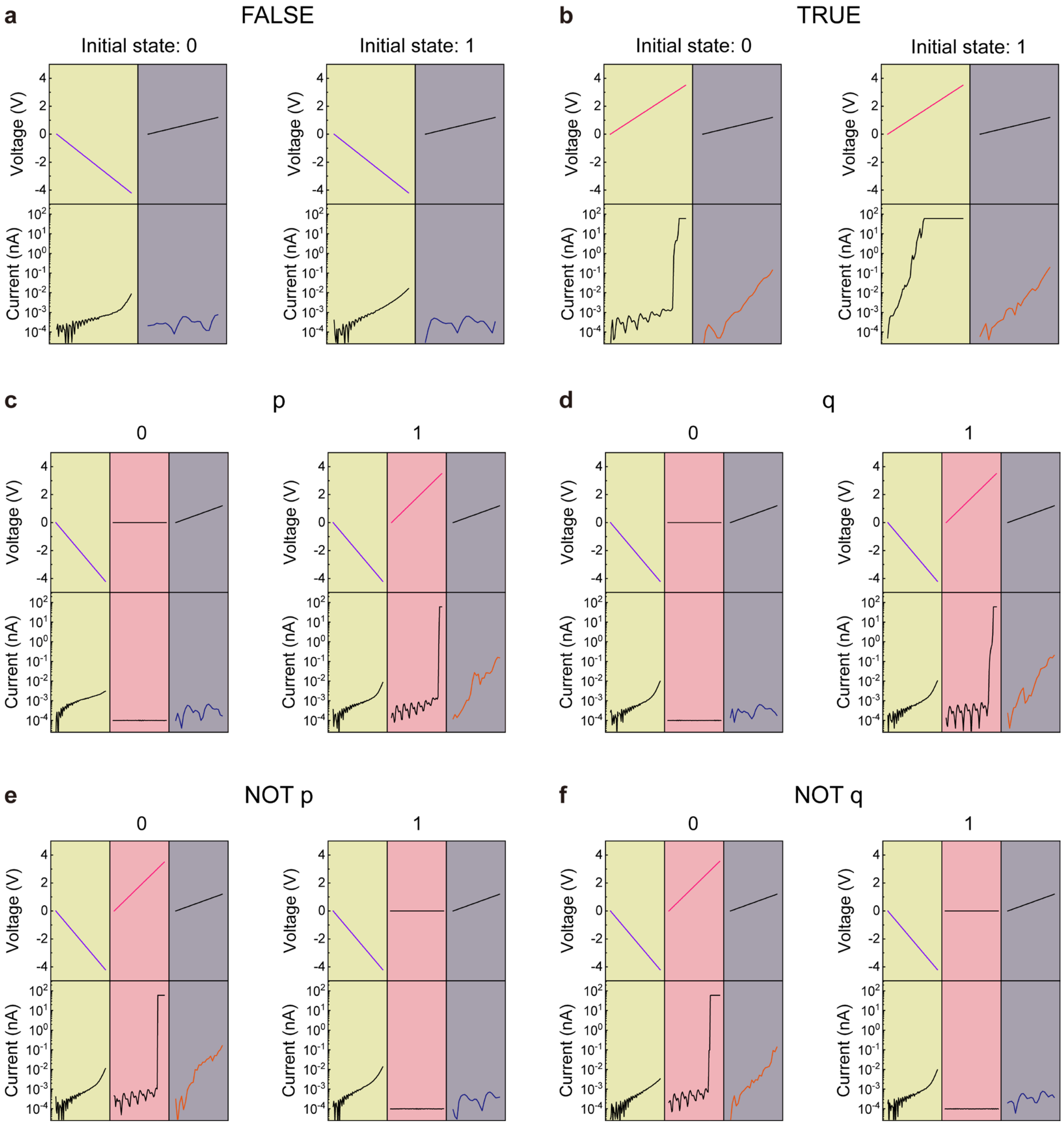

Supplementary Fig. 14 | Experimental demonstration of logic operations. a, FALSE. **b,** TRUE. **c,** *p*. **d,** *q*. **e,** NOT *p*. **f,** NOT *q*.

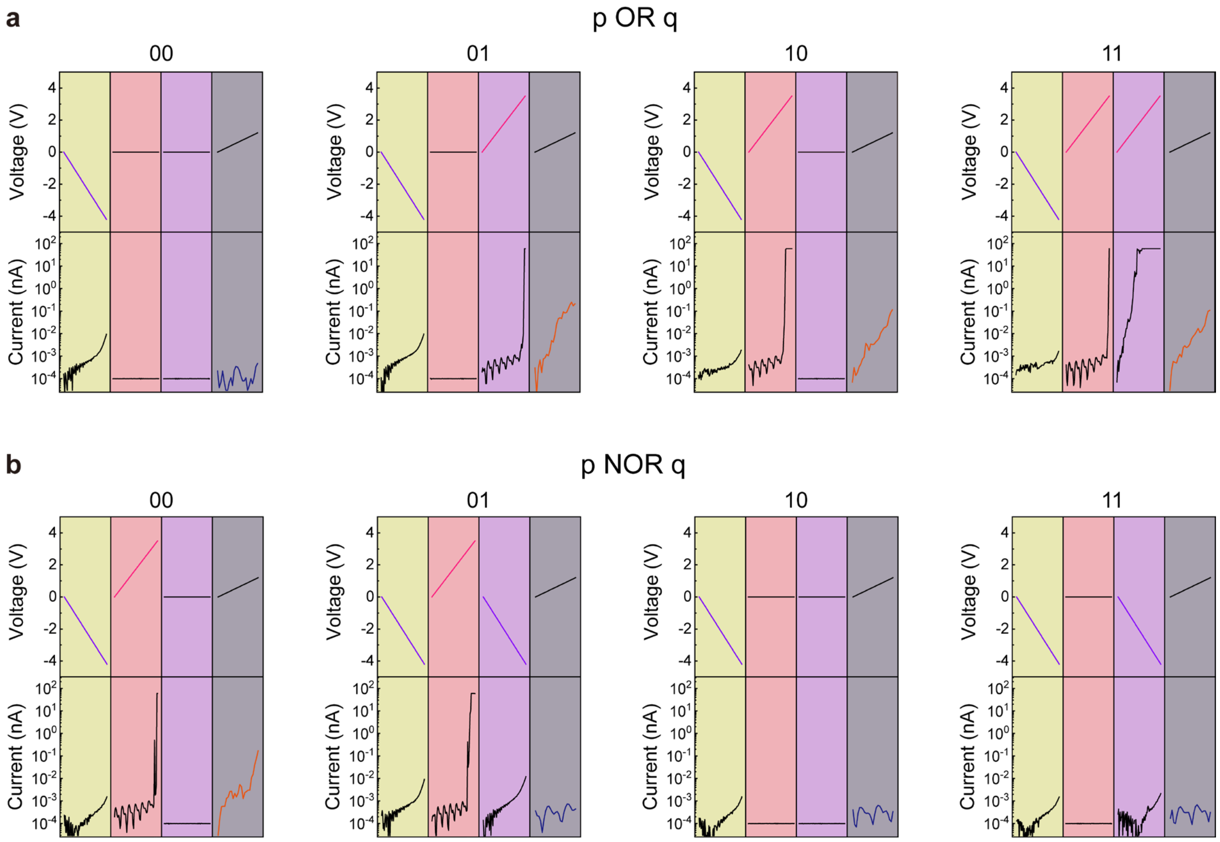

Supplementary Fig. 15 | Experimental demonstration of logic operations. a, *p* OR *q*. **b,** *p* NOR *q*.

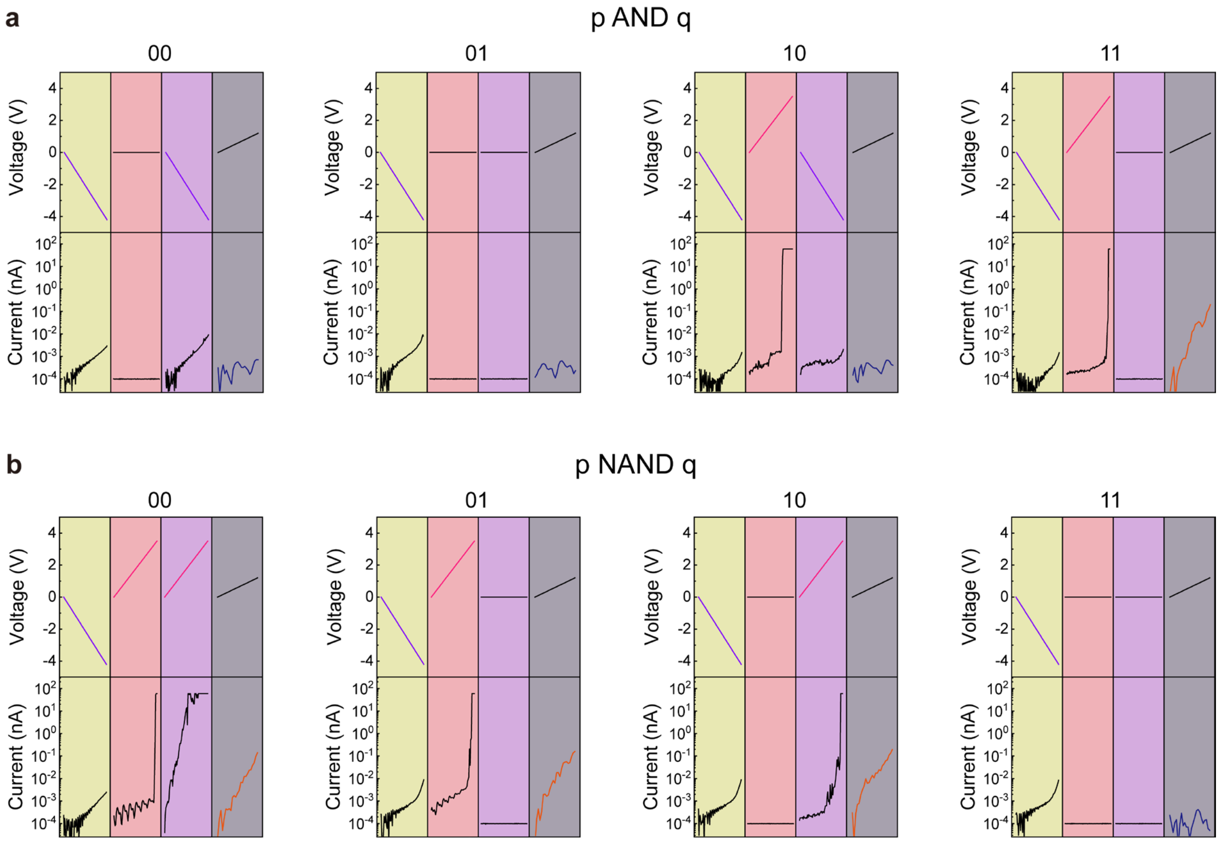

Supplementary Fig. 16 | Experimental demonstration of logic operations. a, *p* AND *q*. ***b*,** *p* NAND *q*.

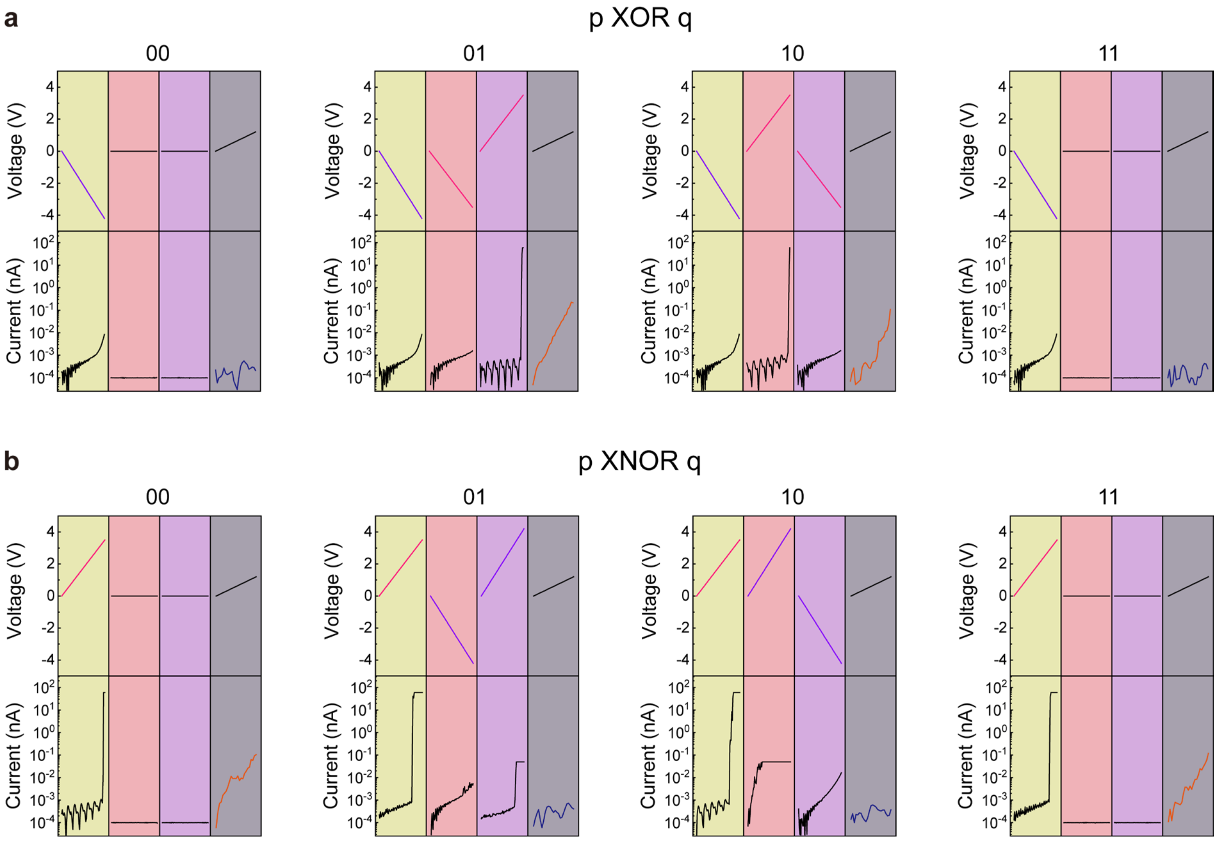

Supplementary Fig. 17 | Experimental demonstration of logic operations. a, *p* XOR *q*. ***b*,** *p* XNOR *q*.

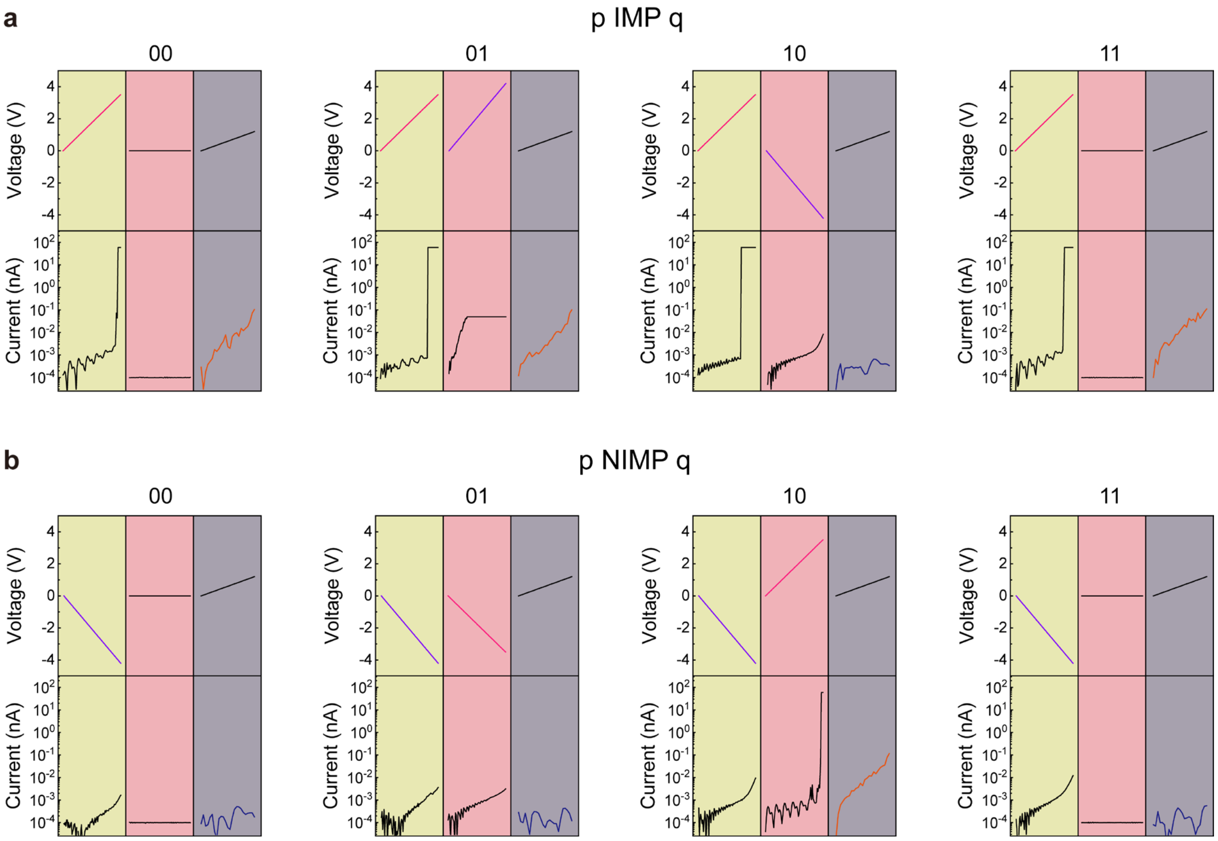

Supplementary Fig. 18 | Experimental demonstration of logic operations. a, *p* IMP *q*. ***b*****,** *p* NIMP *q*.

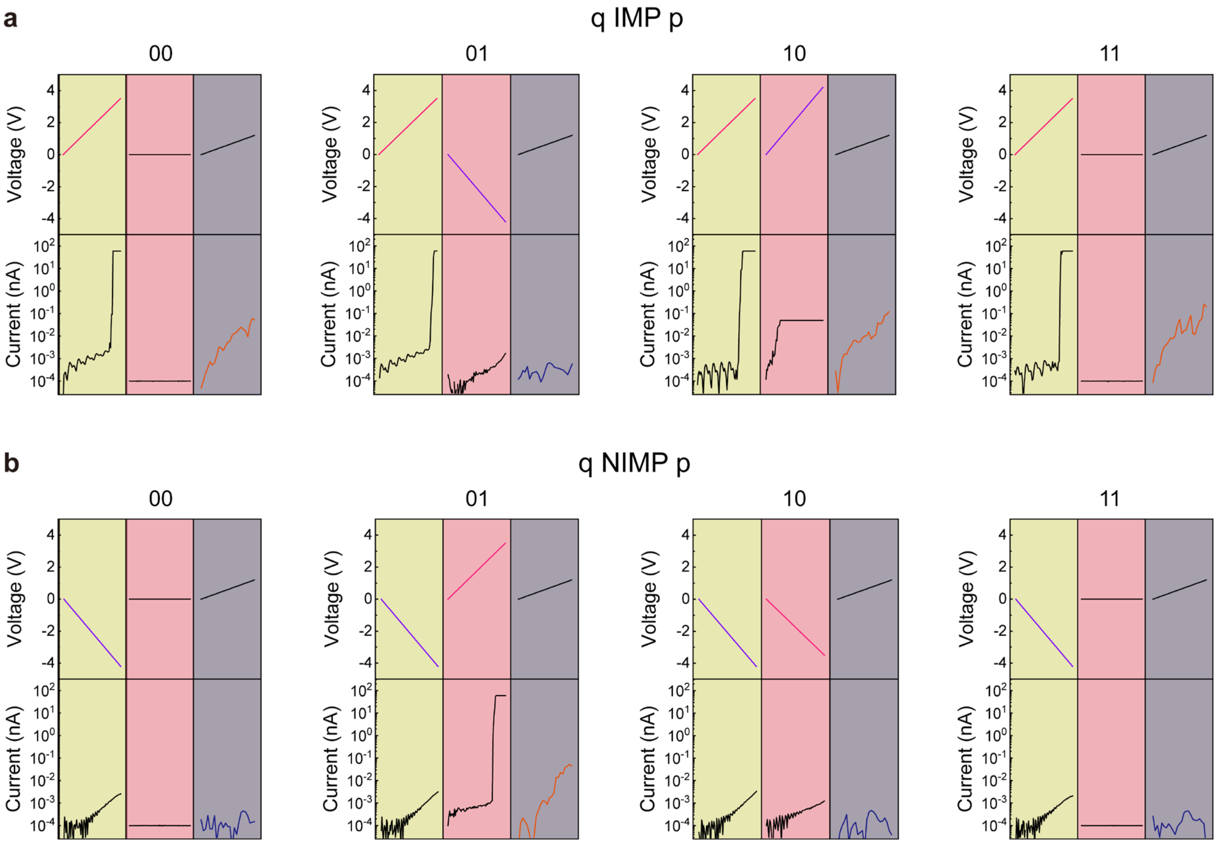

Supplementary Fig. 19 | Experimental demonstration of logic operations. a, *q* IMP *p*. ***b*****,** *q* NIMP *p*.

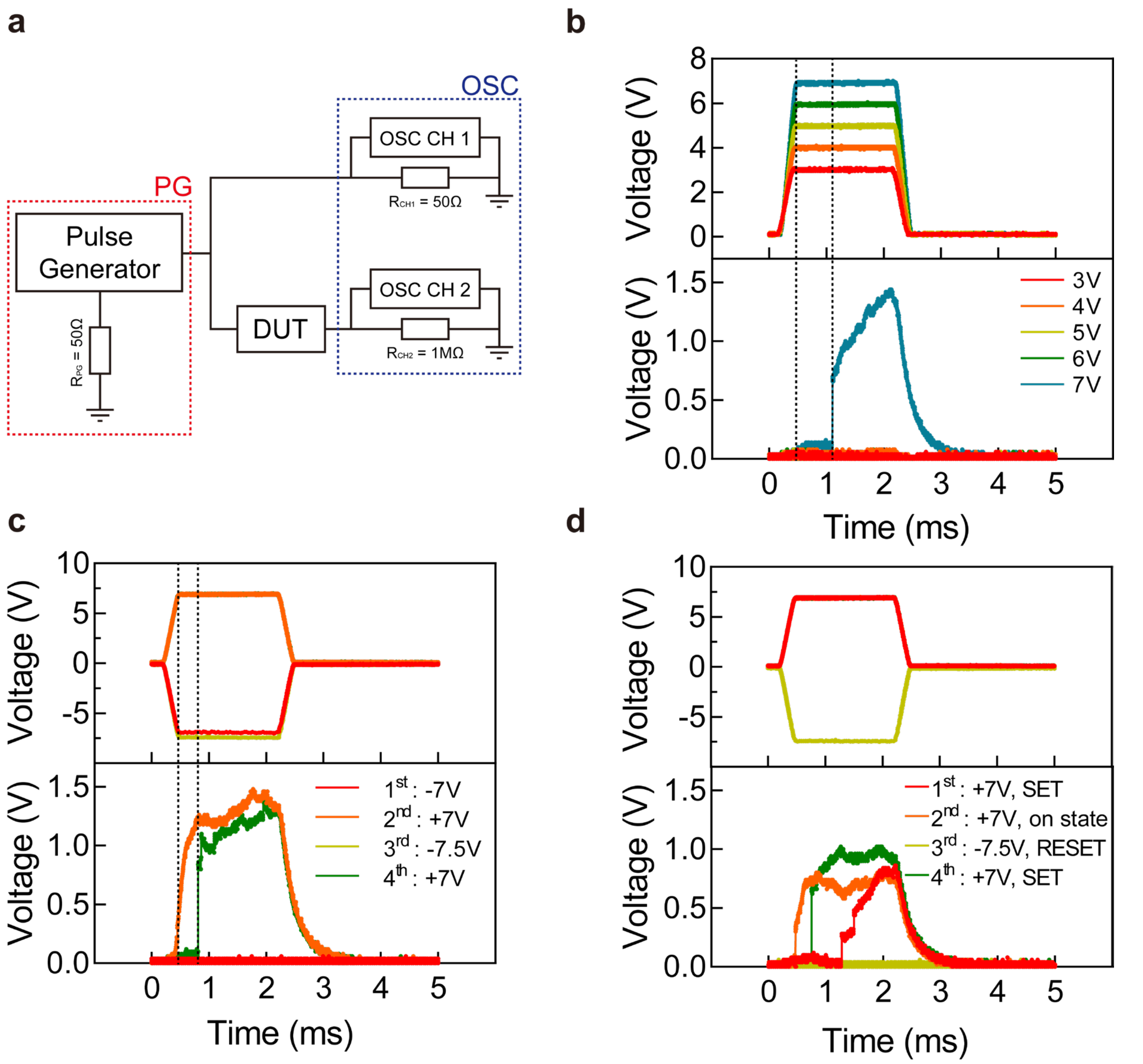

Supplementary Fig. 20 | a, Circuit configuration of the pulse measurement system. Pulse switching of the CuTeHO device at different **b,** SET voltages, and **c,** RESET voltages. **d,** Switching of the device with SET and RESET processes.

Supplementary Note 3. Karnaugh map.

3.1. Karnaugh map of Sum

C_i / AB	**00**	**01**	**11**	**10**
**0**		1		1
**1**	1		1	

Since this Karnaugh map has no adjacent cells, it can be simplified using the Boolean algebra, as follows.

$$S = \overline{A}\overline{B}C_i + \overline{A}B\overline{C_i} + A\overline{B}\overline{C_i} + ABC_i$$

$$= \overline{A}\left(\overline{B}C_i + B\overline{C_i}\right) + A\left(\overline{B}\overline{C_i} + BC_i\right)$$

$$= \overline{A}(B \oplus C_i) + A\left(\overline{B \oplus C_i}\right)$$

$$= (\boldsymbol{A} \oplus \boldsymbol{B}) \oplus \boldsymbol{C_i}$$

3.2. Karnaugh map of Carry-in

C_i / AB	**00**	**01**	**11**	**10**
**0**			1	
**1**		1	1	1

Shaded cells in a carry-in Karnaugh map are grouped together because of the presence of adjacent cells. Also, it can be simplified using the Boolean algebra, as follows.

$$C_o = \overline{A}BC_i + A\overline{B}C_i + AB\overline{C_i} + ABC_i$$

$$= AB + BC_i + AC_i$$

$$= AB + AC_i + BC_i\left(A + \overline{A}\right)$$

$$= ABC_i + AB + AC_i + \overline{A}BC_i$$

$$= AB(1 + C_i) + AC_i + \overline{A}BC_i$$

$$= AB + AC_i + \overline{A}BC_i$$

$$= ABC_i + AB + A\overline{B}C_i + \overline{A}BC_i$$

$$= AB(C_i + 1) + A\overline{B}C_i + \overline{A}BC_i$$

$$= AB + A\overline{B}C_i + \overline{A}BC_i$$

$$= AB + C_i\left(\overline{A}B + A\overline{B}\right)$$

$$= \boldsymbol{AB} + \boldsymbol{C_i}(\boldsymbol{A} \oplus \boldsymbol{B})$$

a

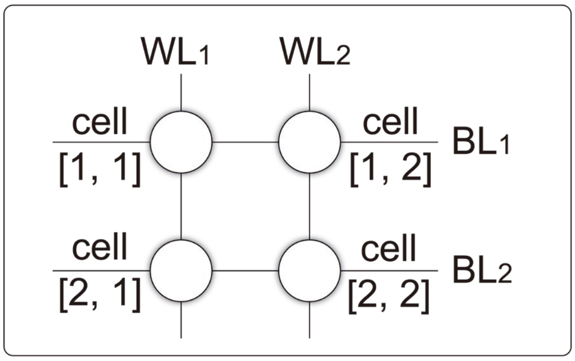

b

A	B	C_i	S	C_o
0	0	0	0	0
0	0	1	1	0
0	1	0	1	0
0	1	1	0	1
1	0	0	1	0
1	0	1	0	1
1	1	0	0	1
1	1	1	1	1

$S = A \oplus B \oplus C_i$

$C_o = A \cdot B + C_i \cdot (A \oplus B)$

c

A	B	B_i	D	B_o
0	0	0	0	0
0	0	1	1	1
0	1	0	1	1
0	1	1	0	1
1	0	0	1	0
1	0	1	0	0
1	1	0	0	0
1	1	1	1	1

$D = A \oplus B \oplus B_i$

$B_o = \overline{A} \cdot B + B_i \cdot \overline{(A \oplus B)}$

Supplementary Fig. 21 | a, 1-bit binary full adder/subtractor in a 2 × 2 memristive crossbar. **b,** Truth table of full adder. The outputs of the 1-bit binary full adder are expressed in XOR, AND, and OR logics. **c,** Truth table of full subtractor. The outputs of the 1-bit binary subtractor are expressed in XOR, AND, and OR logics, similar to the full adder.

Supplementary Note 4. 1-bit binary full adder/subtractor.

4.1. 1-bit binary full adder in a 2 × 2 memristive crossbar.

The Karnaugh map constructed based on the truth table presented in Supplementary Fig. 21 allows for the representation of the output of a 1-bit binary full adder as

$$S = A \oplus B \oplus C_i \tag{S1}$$

$$C_o = A \cdot B + C_i \cdot (A \oplus B) \tag{S2}$$

, where A, B, C_i, S, and C_o are the augend, addend, input-carry, sum, and output-carry, respectively (Eqs. (S1) and (S2) are described in Supplementary Note 3. The logic operations performed in each step and the resulting state changes of the memristors on the 2 × 2 crossbar are summarized in Supplementary Fig. 22. The input is sequentially biased to word lines (WLs) and bitlines (BLs), and the cells are colored after each logic operation. In each step, DC logic operations are performed to satisfy Eqs. (S1) and (S2), including the initialization of the device. Following 8 steps, cell [1, 2] of the 2 × 2 memristive crossbar is mapped to A XOR B XOR C_i, cell [2, 1] to A AND B, and cell [2, 2] to C_i AND (A XOR B). The S can be obtained by simply reading the mapped [1, 2] cell, but the C_o requires an additional OR operation. However, since cells [2, 1] and [2, 2] share BL_2, C_o can be obtained without the OR operation using Kirchhoff's Current Law when a read voltage is applied to WL_1 and WL_2 (Supplementary Fig. 19c). The suggested 1-bit binary full adder utilizes the structural characteristics of the crossbar, demonstrating that the required number of devices and operation steps are lower compared to previously reported memristor-based adders (Supplementary Table 2). Especially, this method is energy efficient because the energy cost is significantly lower compared to the other alternatives.

a

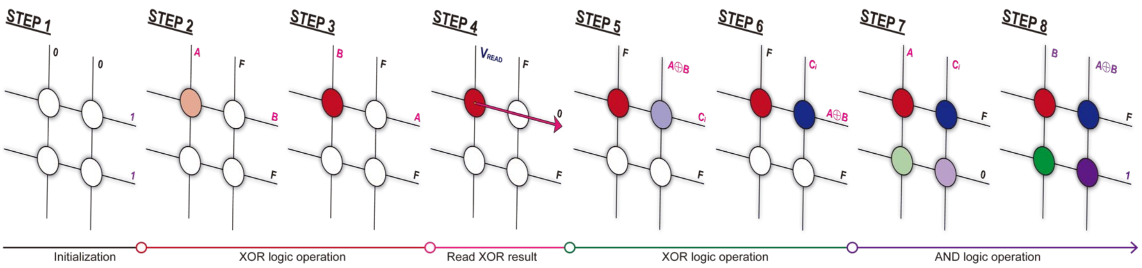

b

	STEP 1			STEP 2			STEP 3			STEP 4		
	WL	BL	status	WL	BL	status	WL	BL	status	WL	BL	status
[1, 1]	0	1	0	A	B	A⊕B(cont.)	B	A	A⊕B	V_{READ}	0	A⊕B
[1, 2]	0	1	0	F	B	0	F	A	0	F	0	0
[2, 1]	0	1	0	A	F	0	B	F	0	V_{READ}	F	0
[2, 2]	0	1	0	F	F	0	F	F	0	F	F	0

	STEP 5			STEP 6			STEP 7			STEP 8		
	WL	BL	status	WL	BL	status	WL	BL	status	WL	BL	status
[1, 1]	F	C_i	A⊕B	F	A⊕B	A⊕B	A	F	A⊕B	B	F	A⊕B
[1, 2]	A⊕B	C_i	A⊕B⊕C_i	C_i	A⊕B	A⊕B⊕C_i	C_i	F	A⊕B⊕C_i	A⊕B	F	A⊕B⊕C_i
[2, 1]	F	F	0	F	F	0	A	0	A · B(cont.)	B	1	A · B
[2, 2]	A⊕B	F	0	C_i	F	0	C_i	0	C_i · (A⊕B) (cont.)	A⊕B	1	C_i · (A⊕B)

c

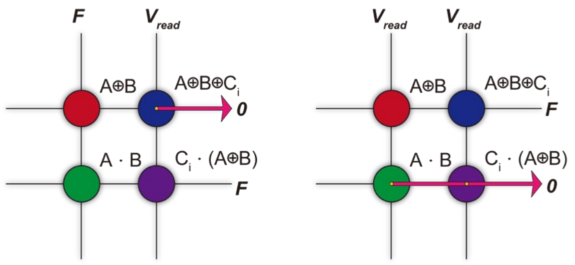

Supplementary Fig. 22 | a, Schematic diagram showing 1-bit binary full adder with 4 memristors in 8 steps. XOR and AND operations are required to determine $\boldsymbol{S}$ and $\boldsymbol{C_o}$. **b,** Detailed operation using two voltage conditions from DC logic. In STEP 1, the reset process is performed to initialize all the cells in HRS. The initialization process in STEP 1 enables the subsequent XOR and AND logic operations to be performed in 2 steps. In STEPs 2-3, the XOR operation of *A* and *B* is performed, and the value obtained from *A* XOR *B* in STEP 4 is used for the $\boldsymbol{S}$ and $\boldsymbol{C_o}$ operations. The $\boldsymbol{C_i}$ and the obtained *A* XOR *B* value are used as inputs to STEPs 5-6, resulting in the output value of $\boldsymbol{S}$ as described in Eq. (3). The AND operation in STEPs 7-8 is performed simultaneously on cells [2, 1] and [2 ,2] as BL_2 is fixed to 0 or 1 and BL_1 is floated. Following 8 steps, cell [1, 2] of the 2 × 2 memristive crossbar is mapped to *A* XOR *B* XOR $\boldsymbol{C_i}$, cell [2, 1] to *A* AND *B*, and cell [2, 2] to $\boldsymbol{C_i}$ AND (*A* XOR *B*). **c,** Readout methods for $\boldsymbol{S}$ and $\boldsymbol{C_o}$.

Supplementary Table 2. Comparison of full adder with memristor-based in-memory computing concepts. The energy cost is defined by the total cells involved during the operating steps[4]. The initialization is excluded for counting both steps and energy costs.

Reference	Fabrication (Device)	Full adder		
		Devices (rows × columns (× layers))	Steps	Energy cost
Huang, P. et al.[5]	Simple ($Al/TiN/Al_2O_3/HfO_x/Pt$)	9 (1 × 9)	10	30
Adam, G. C. et al.[6]	Complex ($Pt/Ti/TiO_{2-x}$ $/Al_2O_3/SiO_2/Pt/Ti/$ $TiO_{2-x}/Al_2O_3/Pt/Ta$)	6 (2 × 2 × 2)	32	51
Cheng, L. et al.[7]	Simple ($Ti/HfO_2/W$)	8 (4 × 2)	27	50
Kim, K. M. et al.[8]	Simple ($TiN//TiO_2/Al$)	3 (1 × 3)	26	28
Xu, L. et al.[9]	Complex ($Pd/TiN/Ta_2O_5/SiO_2/$ $Ta_2O_5/TiN/Pd$)	5 (2 × 3)	7	12
Xu, N. et al.[10]	Complex (Two $Au/Pt/TiO_2/Ti/Pt$ memristors antiparallelly connected)	9 (1 × 5 × 2)	14	28
**This work**	**Simple ($Cu_xTe_{1-x}/HfO_2/Pt$)**	**4 (2 × 2)**	**7**	**9**

4.2. 1-bit binary full subtractor in a 2 × 2 memristive crossbar.

The proposed 1-bit binary full subtractor scheme has a similar procedure to the adder scheme (Supplementary Fig. 23). In STEP 1, the reset process is performed to initialize all the cells in HRS. The initialization process in STEP 1 enables the subsequent XOR and AND logic operations to be performed in 2 steps. In STEPs 2-3, the XOR operation of *A* and *B* is performed, and the value obtained from *A* XOR *B* in STEP 4 is used for the *D* and B_o operations. During STEPs 5-6, an additional XOR operation is performed to obtain the value of *D*, with the input being the negation of *A* XOR *B* that was read in STEP 4. The AND operation in STEPs 7-8 is performed simultaneously on cells [2, 1] and [2, 2] as BL_2 is fixed to 0 or 1 and BL_1 is floated. As before, the negation of *A* and *A* XOR *B* is used as input. Following 8 steps, cell [1, 2] of the 2 × 2 memristive crossbar is mapped to (*A* XOR *B*) XOR B_i, cell [2, 1] to *A* AND *B*, and cell [2, 2] to B_i AND (*A* XOR *B*). As a result of the logic operation, *D* can be obtained by reading the cell [1, 2], and B_o can be obtained by reading WL_1 and WL_2 simultaneously.

a

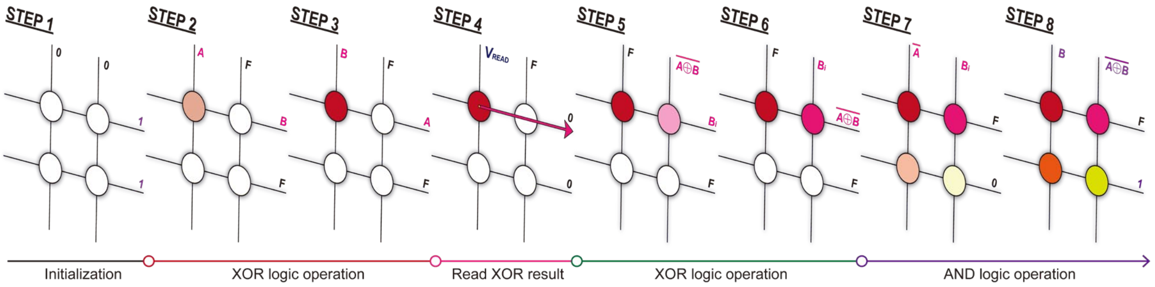

b

	STEP 1			STEP 2			STEP 3			STEP 4		
	WL	BL	status	WL	BL	status	WL	BL	status	WL	BL	status
[1, 1]	0	1	0	A	B	$A\oplus B$ (cont.)	B	A	$A\oplus B$	V_{READ}	0	$A\oplus B$
[1, 2]	0	1	0	F	B	0	F	A	0	F	0	0
[2, 1]	0	1	0	A	F	0	B	F	0	V_{READ}	F	0
[2, 2]	0	1	0	F	F	0	F	F	0	F	F	0

	STEP 5			STEP 6			STEP 7			STEP 8		
	WL	BL	status	WL	BL	status	WL	BL	status	WL	BL	status
[1, 1]	F	B_i	$A\oplus B$	F	$\overline{A\oplus B}$	$A\oplus B$	$\overline{A}$	F	$A\oplus B$	B	F	$A\oplus B$
[1, 2]	$\overline{A\oplus B}$	B_i	$\overline{A\oplus B}\oplus B_i$	B_i	$\overline{A\oplus B}$	$\overline{A\oplus B}\oplus B_i$	B_i	F	$\overline{A\oplus B}\oplus B_i$	$\overline{A\oplus B}$	F	$\overline{A\oplus B}\oplus B_i$
[2, 1]	F	F	0	F	F	0	$\overline{A}$	0	$\overline{A}\cdot B$ (cont.)	B	1	$\overline{A}\cdot B$
[2, 2]	$\overline{A\oplus B}$	F	0	B_i	F	0	B_i	0	$B_i\cdot(\overline{A\oplus B})$ (cont.)	$\overline{A\oplus B}$	1	$B_i\cdot(\overline{A\oplus B})$

c

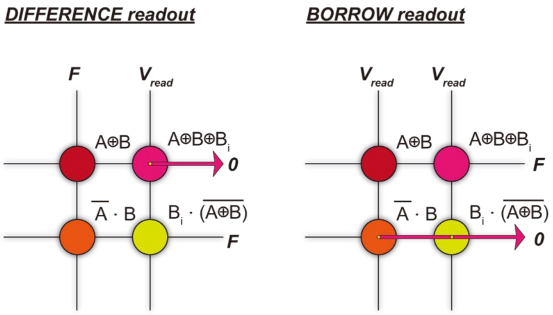

Supplementary Fig. 23 | 1-bit binary full subtractor in a 2 × 2 memristive crossbar. a, Schematic procedure of 1-bit binary full subtractor with 4 memristors in 8 steps. XOR and AND operations are required to determine $\boldsymbol{D}$ and $\boldsymbol{B_o}$. **b,** Detailed operation using two voltage conditions from DC logic. **c,** Readout for $\boldsymbol{D}$ and $\boldsymbol{B_o}$.

4.3. Combined 1-bit full adder-subtractor in a 3 × 2 memristive crossbar.

A combined 1-bit full adder-subtractor can be efficiently implemented in a 3 × 2 memristive crossbar through a singular sequential operation (Supplementary Figs. 24-26). In this scenario, the number of necessary steps is contingent upon the conditions of inputs (augend, addend, carry-in, minuend, subtrahend, and borrow-in), as shown in Supplementary Table 3. In case 1,

when all inputs are identical, executing full adder and full subtractor operations in 10 steps proves more efficient than utilizing two 2 × 2 arrays, with each requiring 8 steps. In case 3, when all inputs are different, the operation requires 16 steps, yet it is advantageous concerning the required number of components. Furthermore, in this configuration, where the adder and subtractor are combined in a single 3 × 2 memristive crossbar, the outputs for both operations can be mapped to the same array without the need for additional cells (Supplementary Fig. 27).

4.3.1. Case 1: All the inputs are identical.

When all the inputs (A_{add}/A_{sub}, B_{add}/B_{sub}, and C_i/B_i) are identical, only two additional AND operations are included for B_o due to the equality between SUM (A_{add} XOR B_{add} XOR C_i) and DIFFERENCE (A_{sub} XOR B_{sub} XOR B_i).

4.3.2. Case 2: Input-carry and input-borrow are different.

In case 2, where inputs A_{add}/A_{sub} and B_{add}/B_{sub} are identical, the sequence remains identical to the previous sequence until STEP 4. However, due to the disparity between C_i and B_i, the operations utilizing these values in the subsequent stages should be carried out independently. By initializing the [1, 1] element of the unused 3 × 2 crossbar after STEP 4, a dedicated storage area is established to preserve independently computed SUM and DIFFERENCE values (STEP 5). By incorporating the 2nd initialization step and including 8 steps for each XOR and AND operation (STEP 6-13), a cumulative sequence of 13 operations is performed to realize the combined 1-bit full adder-subtractor of case 2.

4.3.3. Case 3: All the inputs are different.

When all inputs are different, it is necessary to independently perform operations to acquire the values of A_{add} XOR B_{add} and A_{sub} XOR B_{sub}, which play a crucial role in the SUM and DIFFERENCE logic operations. Consequently, following the reading of results from individual

XOR operations (STEPs 4 and 7), independent logic operations of both SUM and DIFFERENCE are executed, facilitated by the involvement of a 2nd initialization step (STEP 8). During this sequence, a total of 8 steps are carried out. Following this, a sequential logic operation of 8 steps is performed for the operations involving SUM (STEPs 9-10), DIFFERNCE (STEPs 11-12), C_o (STEPs 13-14), and B_o (STEPs 15-16), ultimately leading to the realization of the case 3 combined 1-bit full added-subtractor in 16 steps.

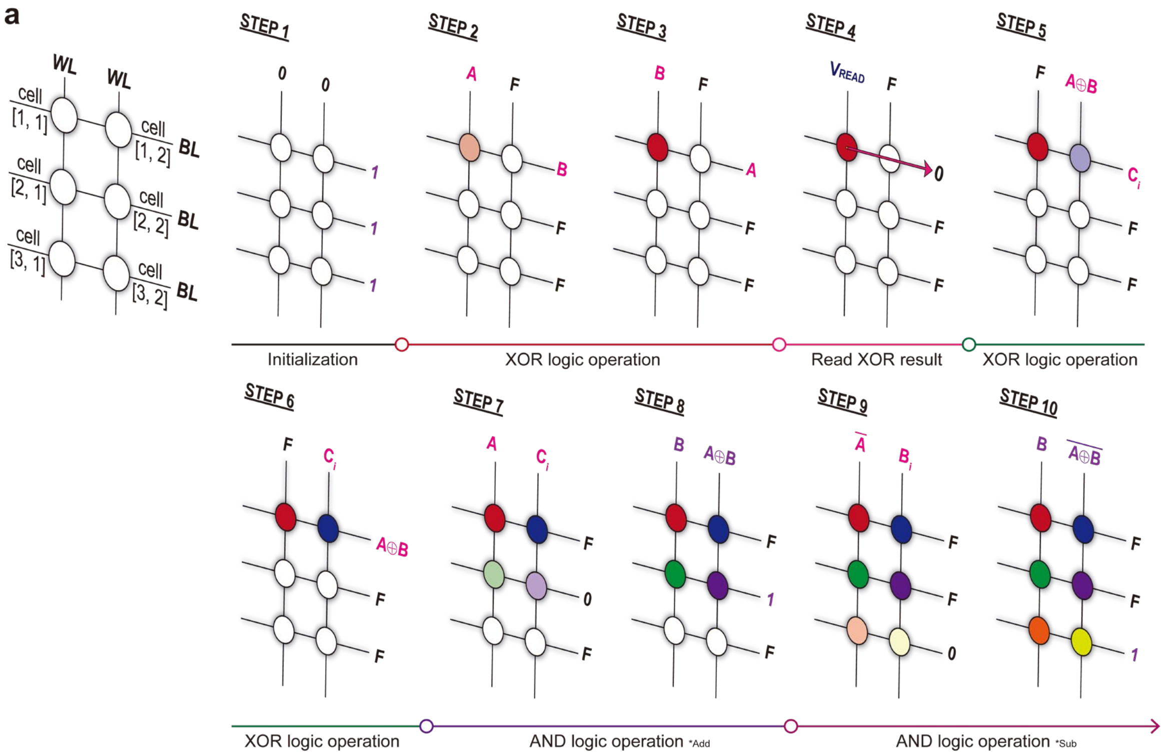

Supplementary Fig. 24 | a, Schematic procedure to perform adding and subtracting operations at once using a 3 × 2 memristive crossbar when all inputs of the adder and subtractor are the same ($\boldsymbol{A_{add}} = \boldsymbol{A_{sub}}$, $\boldsymbol{B_{add}} = \boldsymbol{B_{sub}}$, and $\boldsymbol{C_i} = \boldsymbol{B_i}$) (cont.)

b

	STEP 1			STEP 2			STEP 3			STEP 4		
	WL	BL	status	WL	BL	status	WL	BL	status	WL	BL	status
[1, 1]	0	1	0	A	B	$A \oplus B$ (cont.)	B	A	$A \oplus B$	V_{READ}	0	$A \oplus B$
[1, 2]	0	1	0	F	B	0	F	A	0	F	0	0
[2, 1]	0	1	0	A	F	0	B	F	0	V_{READ}	F	0
[2, 2]	0	1	0	F	F	0	F	F	0	F	F	0
[3, 1]	0	1	0	A	F	0	B	F	0	V_{READ}	F	0
[3, 2]	0	1	0	F	F	0	F	F	0	F	F	0

	STEP 5			STEP 6			STEP 7			STEP 8		
	WL	BL	status	WL	BL	status	WL	BL	status	WL	BL	status
[1, 1]	F	C_i	$A \oplus B$	F	$A \oplus B$	$A \oplus B$	A	F	$A \oplus B$	B	F	$A \oplus B$
[1, 2]	$A \oplus B$	C_i	$A \oplus B \oplus C_i$	C_i	$A \oplus B$	$A \oplus B \oplus C_i$	C_i	F	$A \oplus B \oplus C_i$	$A \oplus B$	F	$A \oplus B \oplus C_i$
[2, 1]	F	F	0	F	F	0	A	0	$A \cdot B$ (cont.)	B	1	$A \cdot B$
[2, 2]	$A \oplus B$	F	0	C_i	F	0	C_i	0	$C_i \cdot (A \oplus B)$ (cont.)	$A \oplus B$	1	$C_i \cdot (A \oplus B)$
[3, 1]	F	F	0	F	F	0	A	F	0	B	F	0
[3, 2]	$A \oplus B$	F	0	C_i	F	0	C_i	F	0	$A \oplus B$	F	0

	STEP 9			STEP 10		
	WL	BL	status	WL	BL	status
[1, 1]	$\overline{A}$	F	$A \oplus B$	B	F	$A \oplus B$
[1, 2]	B_i	F	$A \oplus B \oplus C_i$	$\overline{A \oplus B}$	F	$A \oplus B \oplus C_i$
[2, 1]	$\overline{A}$	F	$A \cdot B$	B	F	$A \cdot B$
[2, 2]	B_i	F	$C_i \cdot (A \oplus B)$	$\overline{A \oplus B}$	F	$C_i \cdot (A \oplus B)$
[3, 1]	$\overline{A}$	0	$\overline{A} \cdot B$ (cont.)	B	1	$\overline{A} \cdot B$
[3, 2]	B_i	0	$B_i \cdot (\overline{A \oplus B})$ (cont.)	$\overline{A \oplus B}$	1	$B_i \cdot (\overline{A \oplus B})$

Supplementary Fig. 24 | b, Detailed operation when the adder and subtractor share the same inputs, allowing the logical operation to be completed in 10 steps (Case 1).

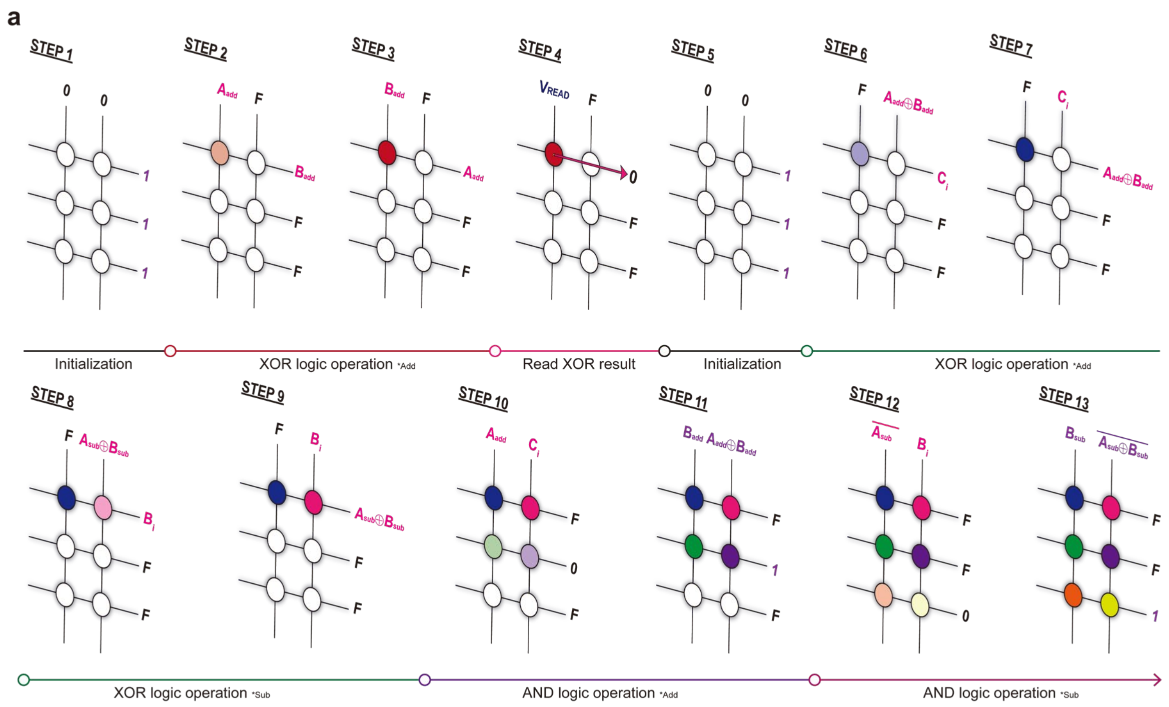

Supplementary Fig. 25 | a, Schematic procedure to perform adding and subtracting operations at once using a 3 × 2 memristive crossbar when input-carry and input-borrow are different ($\boldsymbol{A_{add}} = \boldsymbol{A_{sub}}$, $\boldsymbol{B_{add}} = \boldsymbol{B_{sub}}$, and $\boldsymbol{C_i} \neq \boldsymbol{B_i}$). (cont.)

b

	STEP 1			STEP 2			STEP 3			STEP 4		
	WL	BL	status	WL	BL	status	WL	BL	status	WL	BL	status
[1, 1]	0	1	0	A_{add}	B_{add}	$A_{add}\oplus B_{add}$ (cont.)	B_{add}	A_{add}	$A_{add}\oplus B_{add}$	V_{READ}	0	$A_{add}\oplus B_{add}$
[1, 2]	0	1	0	F	B_{add}	0	F	A_{add}	0	F	0	0
[2, 1]	0	1	0	A_{add}	F	0	B_{add}	F	0	V_{READ}	F	0
[2, 2]	0	1	0	F	F	0	F	F	0	F	F	0
[3, 1]	0	1	0	A_{add}	F	0	B_{add}	F	0	V_{READ}	F	0
[3, 2]	0	1	0	F	F	0	F	F	0	F	F	0

	STEP 5			STEP 6			STEP 7			STEP 8		
	WL	BL	status	WL	BL	status	WL	BL	status	WL	BL	status
[1, 1]	0	1	0	$A_{add}\oplus B_{add}$	C_i	$A_{add}\oplus B_{add}\oplus C_i$	C_i	$A_{add}\oplus B_{add}$	$A_{add}\oplus B_{add}\oplus C_i$	F	B_i	$A_{add}\oplus B_{add}\oplus C_i$
[1, 2]	0	1	0	F	C_i	0	F	$A_{add}\oplus B_{add}$	0	$A_{sub}\oplus B_{sub}$	B_i	$A_{sub}\oplus B_{sub}\oplus B_i$
[2, 1]	0	1	0	$A_{add}\oplus B_{add}$	F	0	C_i	F	0	F	F	0
[2, 2]	0	1	0	F	F	0	F	F	0	$A_{sub}\oplus B_{sub}$	F	0
[3, 1]	0	1	0	$A_{add}\oplus B_{add}$	F	0	C_i	F	0	F	F	0
[3, 2]	0	1	0	F	F	0	F	F	0	$A_{sub}\oplus B_{sub}$	F	0

	STEP 9			STEP 10			STEP 11			STEP 12		
	WL	BL	status	WL	BL	status	WL	BL	status	WL	BL	status
[1, 1]	F	$A_{sub}\oplus B_{sub}$	$A_{add}\oplus B_{add}\oplus C_i$	A_{add}	F	$A_{add}\oplus B_{add}\oplus C_i$	B_{add}	F	$A_{add}\oplus B_{add}\oplus C_i$	$\overline{A_{sub}}$	F	$A_{add}\oplus B_{add}\oplus C_i$
[1, 2]	B_i	$A_{sub}\oplus B_{sub}$	$A_{sub}\oplus B_{sub}\oplus B_i$	C_i	F	$A_{sub}\oplus B_{sub}\oplus B_i$	$A_{add}\oplus B_{add}$	F	$A_{sub}\oplus B_{sub}\oplus B_i$	B_i	F	$A_{sub}\oplus B_{sub}\oplus B_i$
[2, 1]	F	F	0	A_{add}	0	$A_{add}\cdot B_{add}$ (cont.)	B_{add}	1	$A_{add}\cdot B_{add}$	$\overline{A_{sub}}$	F	$A_{add}\cdot B_{add}$
[2, 2]	B_i	F	0	C_i	0	$C_i\cdot(A_{add}\oplus B_{add})$ (cont.)	$A_{add}\oplus B_{add}$	1	$C_i\cdot(A_{add}\oplus B_{add})$	B_i	F	$C_i\cdot(A_{add}\oplus B_{add})$
[3, 1]	F	F	0	A_{add}	F	0	B_{add}	F	0	$\overline{A_{sub}}$	0	$\overline{A_{sub}}\cdot B_{sub}$ (cont.)
[3, 2]	B_i	F	0	C_i	F	0	$A_{add}\oplus B_{add}$	F	0	B_i	0	$B_i\cdot\overline{(A_{sub}\oplus B_{sub})}$ (cont.)

	STEP 13		
	WL	BL	status
[1, 1]	B_{sub}	F	$A_{add}\oplus B_{add}\oplus C_i$
[1, 2]	$\overline{A_{sub}\oplus B_{sub}}$	F	$A_{sub}\oplus B_{sub}\oplus B_i$
[2, 1]	B_{sub}	F	$A_{add}\cdot B_{add}$
[2, 2]	$\overline{A_{sub}\oplus B_{sub}}$	F	$C_i\cdot(A_{add}\oplus B_{add})$
[3, 1]	B_{sub}	1	$\overline{A_{sub}}\cdot B_{sub}$
[3, 2]	$\overline{A_{sub}\oplus B_{sub}}$	1	$B_i\cdot\overline{(A_{sub}\oplus B_{sub})}$

Supplementary Fig. 25 | b, Detailed operation when the adder and subtractor share identical inputs, except for $\boldsymbol{B_i}$ and $\boldsymbol{C_i}$, allowing the logical operations to be accomplished in 13 steps (Case 2).

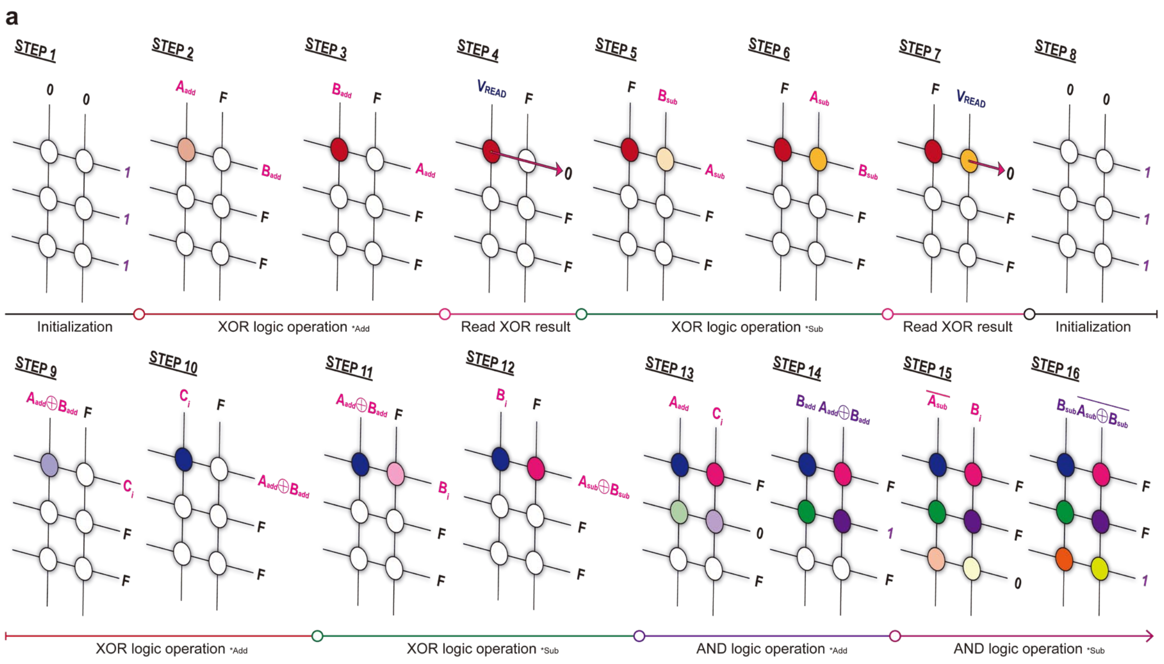

Supplementary Fig. 26 | a, Schematic procedure to perform adding and subtracting operations at once using a 3 × 2 memristive crossbar when all inputs of adder and subtractor are different ($\boldsymbol{A_{add}} \neq \boldsymbol{A_{sub}}$, $\boldsymbol{B_{add}} \neq \boldsymbol{B_{sub}}$, and $\boldsymbol{C_i} \neq \boldsymbol{B_i}$). (cont.)

b

	STEP 1			STEP 2			STEP 3			STEP 4		
	WL	BL	status	WL	BL	status	WL	BL	status	WL	BL	status
[1, 1]	0	1	0	A_{add}	B_{add}	$A_{add}\oplus B_{add}$ (cont.)	B_{add}	A_{add}	$A_{add}\oplus B_{add}$	V_{READ}	0	$A_{add}\oplus B_{add}$
[1, 2]	0	1	0	F	B_{add}	0	F	A_{add}	0	F	0	0
[2, 1]	0	1	0	A_{add}	F	0	B_{add}	F	0	V_{READ}	F	0
[2, 2]	0	1	0	F	F	0	F	F	0	F	F	0
[3, 1]	0	1	0	A_{add}	F	0	B_{add}	F	0	V_{READ}	F	0
[3, 2]	0	1	0	F	F	0	F	F	0	F	F	0

	STEP 5			STEP 6			STEP 7			STEP 8		
	WL	BL	status	WL	BL	status	WL	BL	status	WL	BL	status
[1, 1]	F	B_{sub}	$A_{add}\oplus B_{add}$	F	A_{sub}	$A_{add}\oplus B_{add}$	F	0	$A_{add}\oplus B_{add}$	0	1	0
[1, 2]	A_{sub}	B_{sub}	$A_{sub}\oplus B_{sub}$ (cont.)	B_{sub}	A_{sub}	$A_{sub}\oplus B_{sub}$	V_{READ}	0	$A_{sub}\oplus B_{sub}$	0	1	0
[2, 1]	F	F	0	F	F	0	F	F	0	0	1	0
[2, 2]	A_{sub}	F	0	B_{sub}	F	0	V_{READ}	F	0	0	1	0
[3, 1]	F	F	0	F	F	0	F	F	0	0	1	0
[3, 2]	A_{sub}	F	0	B_{sub}	F	0	V_{READ}	F	0	0	1	0

	STEP 9			STEP 10			STEP 11			STEP 12		
	WL	BL	status	WL	BL	status	WL	BL	status	WL	BL	status
[1, 1]	$A_{add}\oplus B_{add}$	C_i	$A_{add}\oplus B_{add}\oplus C_i$	C_i	$A_{add}\oplus B_{add}$	$A_{add}\oplus B_{add}\oplus C_i$	F	B_i	$A_{add}\oplus B_{add}\oplus C_i$	F	$A_{sub}\oplus B_{sub}$	$A_{add}\oplus B_{add}\oplus C_i$
[1, 2]	F	C_i	0	F	$A_{add}\oplus B_{add}$	0	$A_{sub}\oplus B_{sub}$	B_i	$A_{sub}\oplus B_{sub}\oplus B_i$	B_i	$A_{sub}\oplus B_{sub}$	$A_{sub}\oplus B_{sub}\oplus B_i$
[2, 1]	$A_{add}\oplus B_{add}$	F	0	C_i	F	0	F	F	0	F	F	0
[2, 2]	F	F	0	F	F	0	$A_{sub}\oplus B_{sub}$	F	0	B_i	F	0
[3, 1]	$A_{add}\oplus B_{add}$	F	0	C_i	F	0	F	F	0	F	F	0
[3, 2]	F	F	0	F	F	0	$A_{sub}\oplus B_{sub}$	F	0	B_i	F	0

	STEP 13			STEP 14			STEP 15			STEP 16		
	WL	BL	status	WL	BL	status	WL	BL	status	WL	BL	status
[1, 1]	A_{add}	F	$A_{add}\oplus B_{add}\oplus C_i$	B_{add}	F	$A_{add}\oplus B_{add}\oplus C_i$	$\overline{A_{sub}}$	F	$A_{add}\oplus B_{add}\oplus C_i$	B_{sub}	F	$A_{add}\oplus B_{add}\oplus C_i$
[1, 2]	C_i	F	$A_{sub}\oplus B_{sub}\oplus B_i$	$A_{add}\oplus B_{add}$	F	$A_{sub}\oplus B_{sub}\oplus B_i$	B_i	F	$A_{sub}\oplus B_{sub}\oplus B_i$	$\overline{A_{sub}\oplus B_{sub}}$	F	$A_{sub}\oplus B_{sub}\oplus B_i$
[2, 1]	A_{add}	0	$A_{add}\cdot B_{add}$ (cont.)	B_{add}	1	$A_{add}\cdot B_{add}$	$\overline{A_{sub}}$	F	$A_{add}\cdot B_{add}$	B_{sub}	F	$A_{add}\cdot B_{add}$
[2, 2]	C_i	0	$C_i\cdot(A_{add}\oplus B_{add})$ (cont.)	$A_{add}\oplus B_{add}$	1	$C_i\cdot(A_{add}\oplus B_{add})$	B_i	F	$C_i\cdot(A_{add}\oplus B_{add})$	$\overline{A_{sub}\oplus B_{sub}}$	F	$C_i\cdot(A_{add}\oplus B_{add})$
[3, 1]	A_{add}	F	0	B_{add}	F	0	$\overline{A_{sub}}$	0	$\overline{A_{sub}}\cdot B_{sub}$ (cont.)	B_{sub}	1	$\overline{A_{sub}}\cdot B_{sub}$
[3, 2]	C_i	F	0	$A_{add}\oplus B_{add}$	F	0	B_i	0	$B_i\cdot\overline{(A_{sub}\oplus B_{sub})}$ (cont.)	$\overline{A_{sub}\oplus B_{sub}}$	1	$B_i\cdot\overline{(A_{sub}\oplus B_{sub})}$

Supplementary Fig. 26 | b, Detailed operation when the adder and subtractor have different inputs, allowing the logical operation to be completed in 16 steps (Case 3).

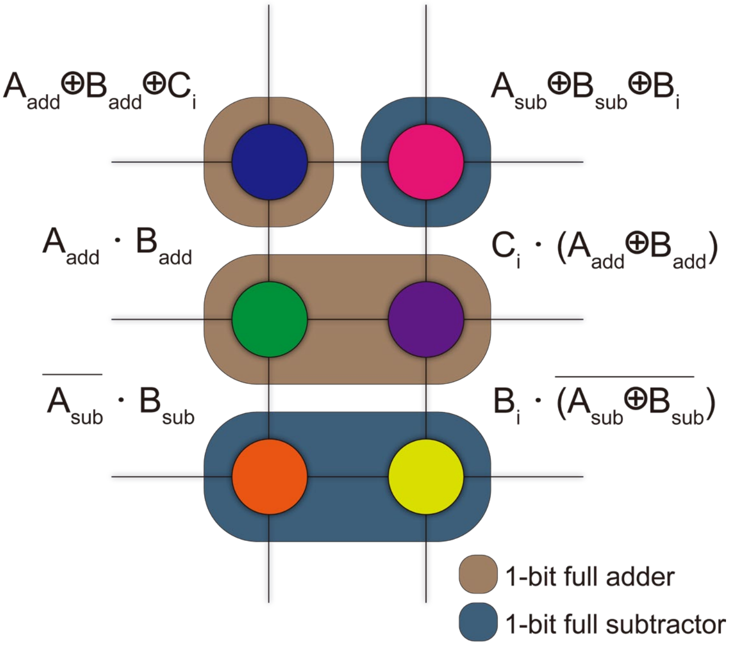

Supplementary Fig. 27 | 1-bit binary adder-subtractor in a 3 × 2 memristive crossbar that allows addition and subtraction.

Supplementary Table 3. Required number of steps and cells for different operations.

Operation type	Number of steps	Number of cells (crossbar size)	Conditions
**1-bit full adder**	8	4 (2 × 2)	-
**1-bit full subtractor**	8	4 (2 × 2)	-
**Combined 1-bit full adder - subtractor**	10	6 (2 × 3)	*Case 1*<br>$A_{add} = A_{sub}$<br>$B_{add} = B_{sub}$<br>$C_i = B_i$
	13	6 (2 × 3)	*Case 2*<br>$A_{add} = A_{sub}$<br>$B_{add} = B_{sub}$<br>$C_i \neq B_i$
	16	6 (2 × 3)	*Case 3*<br>$A_{add} \neq A_{sub}$<br>$B_{add} \neq B_{sub}$<br>$C_i \neq B_i$

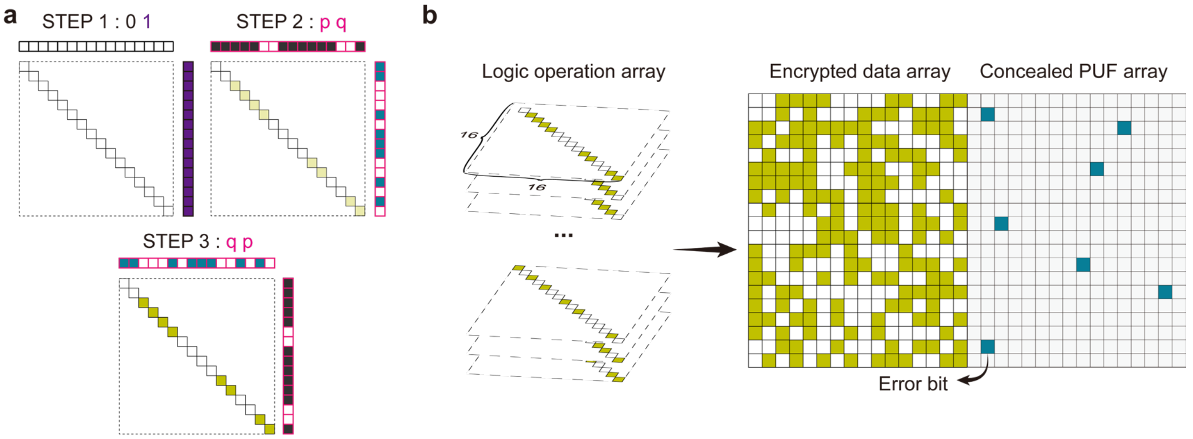

Supplementary Fig. 28 | a, Demonstration of the encryption from the hybrid memory-PUF array with 3-step XOR DC logic. **b,** Memory-PUF array that the letter-shaped data is replaced with encrypted data through the logic operation array, and the PUF data is partially reset and concealed. This operation makes the memory-PUF array fully encrypted.

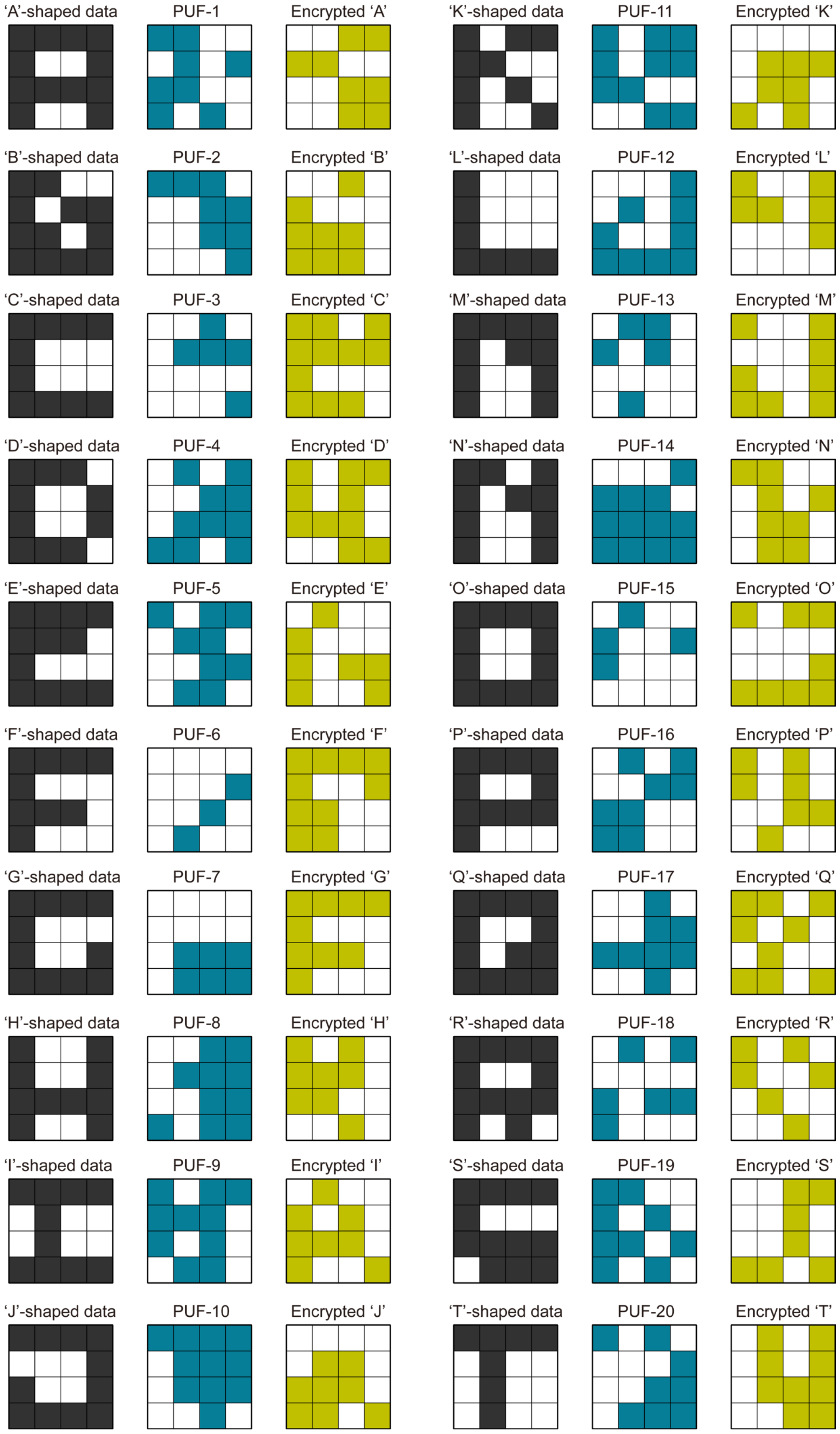

Supplementary Fig. 29 | Encryption of 20 16-bit letter-shaped data ('A' to 'T') with cryptographic keys (PUF-1 to PUF-20) obtained in Supplementary Fig. 8.

Supplementary Note 5. The hardware setup for a fully encrypted memory-PUF implementation.

A fully encrypted memory-PUF implementation requires a circuit structure that stores information in the crossbar and allows for logic operations. In the proposed circuit system, the selection of the crossbar cell is implemented with a multiplexer (MUX) controlled from a microcontroller unit (MCU), as illustrated in Supplementary Fig. 30. The MCU can also be used to apply the voltage required for general data storage, as well as the median SET voltage for the PUF and the voltage required for two conditions for logic operations. The high nonlinearity and self-rectifying characteristics of the CuTeHO memristor remove the requirement for a selector device in crossbar configuration. However, during the write operations, a 1/2 voltage scheme can be employed to reduce the unwanted impact caused by cells that share word lines and bit lines with the selected cell. The read and write modes are controlled by an independent switch, and the voltage amplitude is adjusted through a buffer and 1/2-gain inverting amplifier configuration. When the read mode switch is activated, the BL of the selected cell is connected to a transimpedance amplifier (TIA), and the current is sensed using the MCU's ADC. The DC logic operation suggested in the main text should not only be performed in a separate array but should be able to apply voltage to both WLs and BLs. In logic mode, when the write mode is activated, the BL DAC is interconnected with the BL of the logic operation array, providing the capability of independent voltage biasing. Similar to the memory-PUF array, when the read mode switch is activated, the selected cell's BL within the array is connected to the TIA, enabling the sequential reading of the results of logic operations stored in the diagonal components through the MCU's ADC. The current information sampled by the ADC can be stored in the buffer memory of the MCU and utilized to replace pre-existing data with new data derived from logic operations.

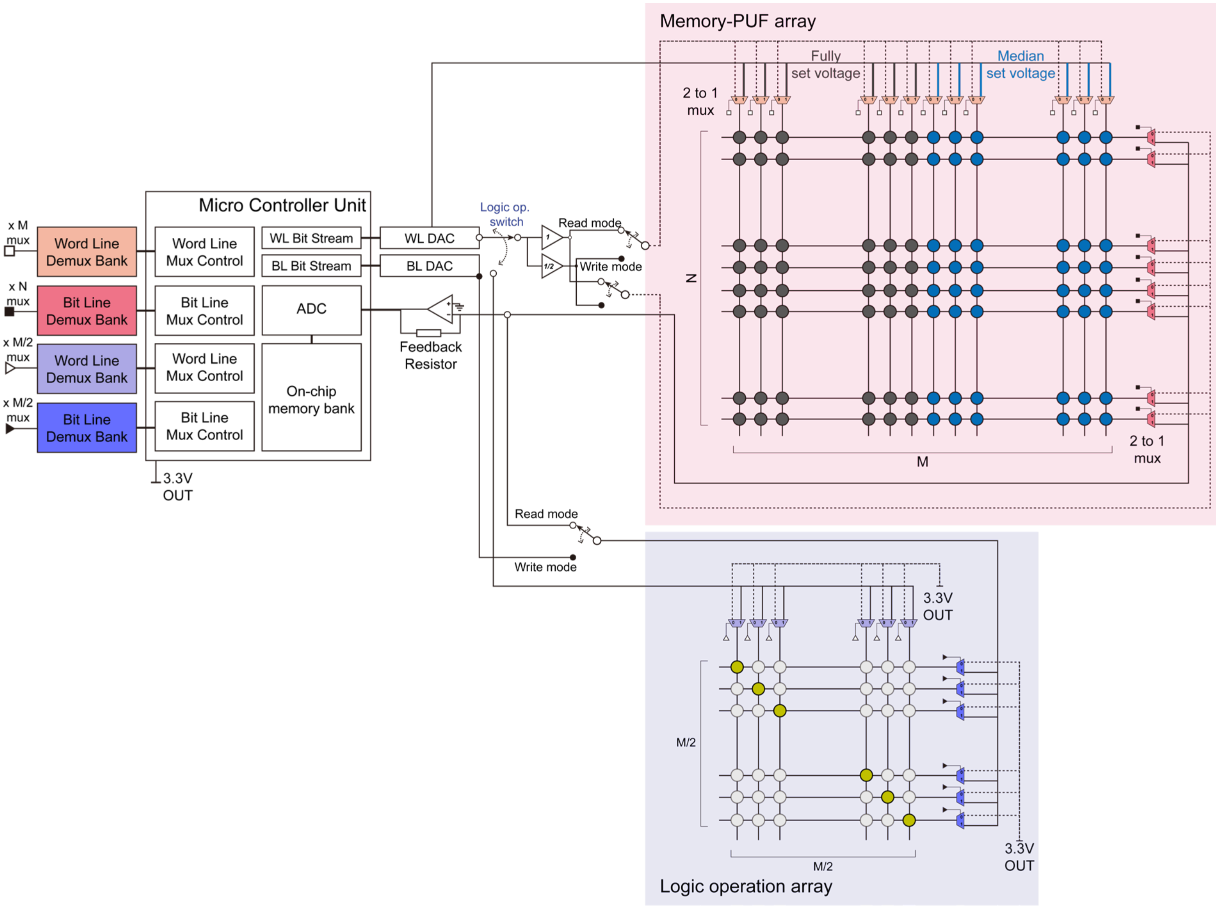

Supplementary Fig. 30 | Schematic diagram of the hardware setup for a fully encrypted memory-PUF implementation.

Supplementary Note 6. Encrypted inference.

Following the demonstration of generating the cryptographic keys and data encryption using such keys, both using the CuTeHO memristors, an entire AI inference process was performed on the encrypted data (Supplementary Figs. 31a). The demonstration of encrypted inference as a primitive form of homomorphic encryption was performed on a multi-memristor system and evaluated the ability of the encryption scheme in faithfully maintaining the underlying data. For this demonstration, a set of 64 × 64 memristor crossbar arrays, constructed using CMOS-integrated memristors designed for vector-matrix multiplications (Supplementary Figs. 31b-d), described elsewhere[11], was used.

The generation of the PUF (key) was performed on the CuTeHO memristors, while the encryption (by simple addition), followed by inference (consisting of vector-matrix multiplications or VMM), and decryption (by simple subtraction) were performed on oxide memristors designed for in-memory vector-matrix multiplications, consisting of 64 × 64 memristor crossbars, described elsewhere in detail[11]. The memristor consists of a memory layer (TaO_x and Ta) and platinum (Pt) electrodes. Simple addition and subtraction were achieved by encoding the vectors to be added (or subtracted) onto two rows of a memristor crossbar and performing VMM on the two stored rows of information by using inputs [+1, +1] for addition and [+1, -1] for subtraction. The all-memristor implementation, especially on the VMM chip, included fully integrated sensing, driving, amplifying and analog-digital conversion circuits, which were interfaced with a control PCB, controlled via a Raspberry-pi system.

The weights required for the inference process were obtained via a technique known as memristor activity-difference energy minimization (MADEM), which was introduced by us recently[12]. The technique employed a memristor-crossbar-based hardware as a part of the training process, wherein the VMM was performed on the memristor crossbars, while other functions (neuron activation, intermediate state storage, etc.) were implemented in software. The technique is based on a numerical training algorithm (unlike backpropagation, which is an

analytical algorithm). It is specifically suited to be operated on memristor hardware, which is typically probabilistic and poses uncertainty issues (errors, noise, asymmetry, variability, etc.). Similar to the training process, the inference process also offloaded any non-VMM operation (e.g., neuron functions) to be implemented in software. Two different tasks were investigated: reconstruction of corrupted words using a 1-layer neural network, and classification of words using a 2-layer neural network.

The CuTeHO memristor crossbar was first used to perform a simple additive encryption process, where input user data A is encrypted into $A^* = A + k$, using key k. Then, A^* was fed into a trained neural network with weights W, resulting in a dot product output $A^*{\cdot}W$. A two-layer network (24 input, 33 hidden, and 7 output neurons) was employed, which was trained to classify seven distorted braille words (Supplementary Figs. 31e and f). Identical weights trained in the previous work in the Hopfield network structure[12] were deployed for this encrypted pattern classification. The output of the neural network was then decrypted into $A{\cdot}W$ via $A{\cdot}W = A^*{\cdot}W - k{\cdot}W$. The result $A{\cdot}W$ is used to infer the desired quantity. The process of finding A^* involved multiple VMMs followed by neuron functions. To maintain the simplicity of the encryption process, we performed a decryption before any neuron function. Although there are several fully homomorphic encryption functions, which enable a wide range of mathematical operations on encrypted data, including neuron activation, those functions are typically complex, and implementation of such functions (especially on post-digital hardware) would be a study of their own.

The inference accuracies with and without encryption were measured (Supplementary Fig. 31g). A lower maximum conductance reduces leakage currents and power consumption, which was investigated by lowering the range of conductances. Below ~ 40 μS, the accuracies with encrypted and unencrypted data are generally lower than those above 40 μS, with the encrypted inference accuracy being measurably lower than the unencrypted inference accuracy. However,

above 40 μS, no significant difference was observed between the encrypted and unencrypted accuracies, indicating that encryption did not degrade the accuracy by a measurable quantity under suitable operating conditions (Supplementary Fig. 32). Similar results were observed in the reconstruction task of Braille representations (Supplementary Fig. 33). This demonstration is a primitive form of homomorphic encryption, which aims to preserve privacy of user data and operates only on encrypted data. These principles may be used in the future for federated learning and homomorphic-encryption-based training systems (Supplementary Fig. 34 and Supplementary Note 7), wherein not only AI inference but the entire training process happens on encrypted data. Although this demonstration did not incorporate a rigorous encryption scheme or sophisticated data processing techniques, this minimalistic mathematical process illustrates that the encryption scheme enables reliable processing and recovery of the underlying data despite the probabilistic nature of the data processing technique (i.e., using analog hardware).

Overall, this work shows that, first, it is possible to perform end-to-end memristor-based security + logic computing; second, minimal to no loss of accuracy has incurred in doing so, while energy is reduced and speed is increased. Neither of the observations is a trivial expectation. The first observation requires memristors to exhibit both highly probabilistic (essentially stochastic in this case) behavior for encryption and highly deterministic behavior for logic. The second observation is counterintuitive because memristor-based computing has largely failed to compete with prevailing digital computers because of its uncertainties (errors, noise, asymmetry, variability, etc.), which are known to lead to low accuracies. This work shows that high accuracy can be maintained by clever exploitation of such uncertainties in certain operating conditions and by mitigating such uncertainties in other operating conditions via clever algorithms. It is emphasized that in most AI, probabilistic computing is not expected to outperform digital hardware running digital-friendly algorithms such as backpropagation in

terms of algorithmic superiority, but as this work has demonstrated, significant gains in overall energy expenditure to achieve the same result are expected.

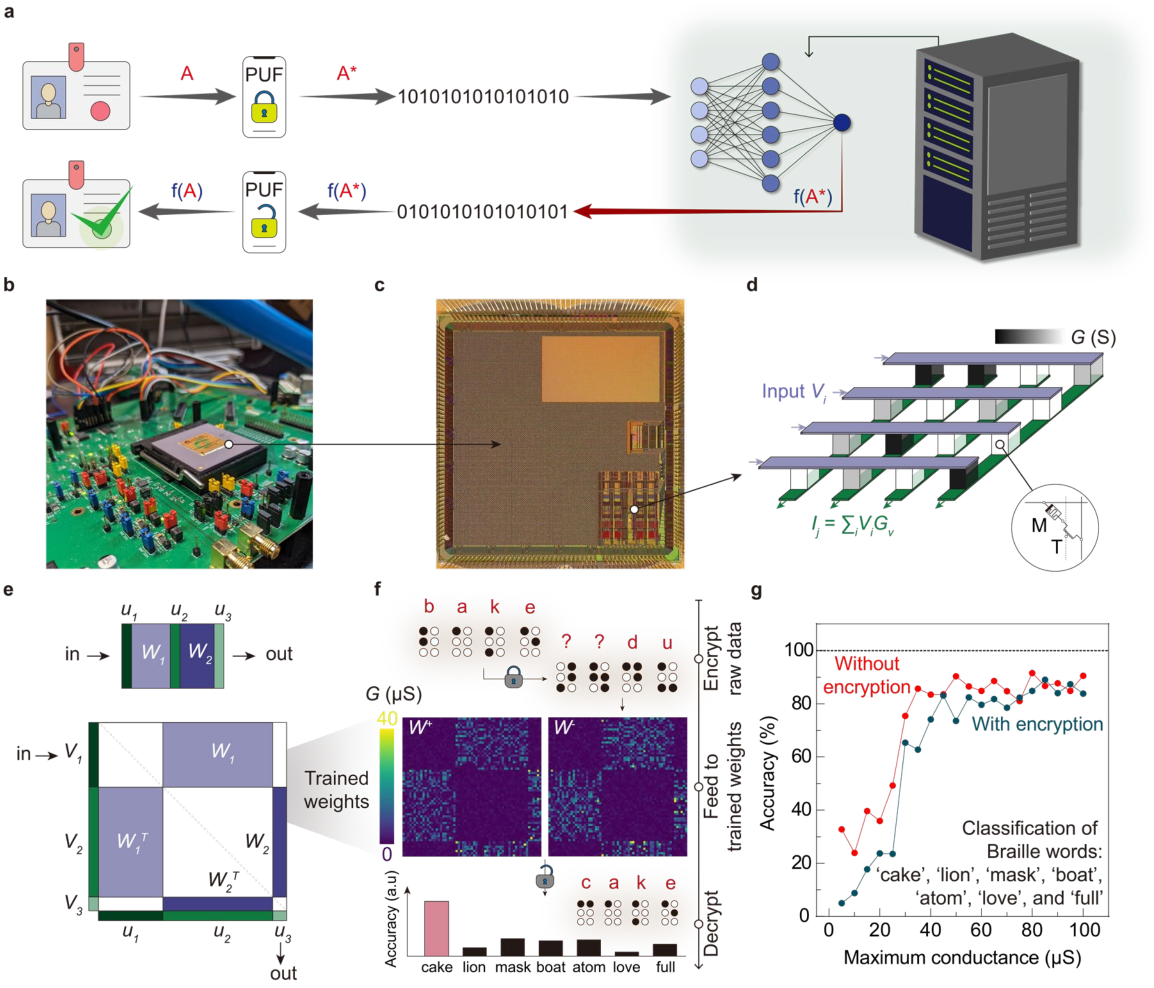

Supplementary Fig. 31 | a, Encrypted inference framework. **b**, The system (circuit board and chip) used for AI encryption. **c**, Photograph of the custom-designed fully integrated memristor system-on-chip die. **d**, Illustration of the memristor crossbar within the chip. **e**, A two-layer neural network, composed of two original matrices for the weights of two layers and two transposed matrices to implement an activity-difference AI inference technique. **f**, Experimental conductance values of positive and negative weights after training, used for classifying the Braille representations. Illustration of the encryption of the Braille representation of 'bake' (user input). The output classification of the input as 'cake'. **g**, Classification accuracies with and without encryption.

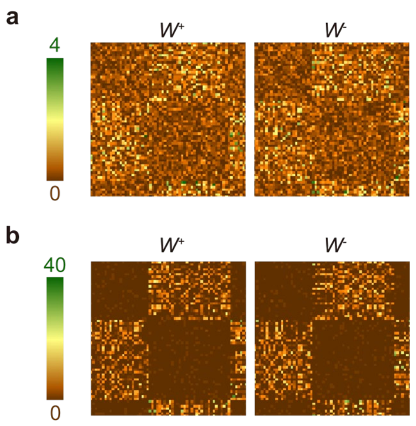

Supplementary Fig. 32 | Programming of the trained weights with two different maximum conductance values. a, Maximum conductance of 4 µS, and **b,** 40 µS for a 2-layered neural network. The matrix programmed with a maximum conductance of 4 µS is noisier, leading to worse results.

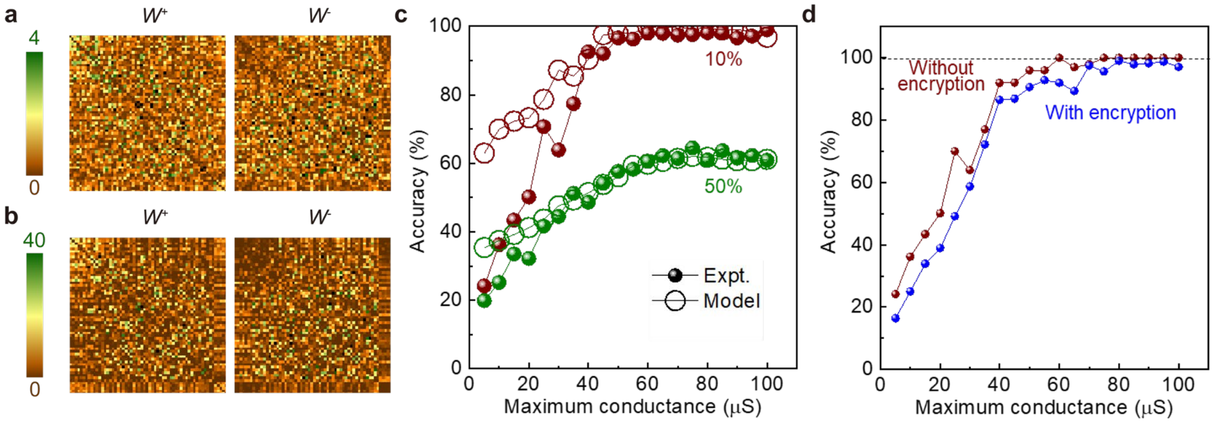

Supplementary Fig. 33 | 1-layer neural network used in encrypted inference. Programming of the trained weights with two different maximum conductance values of **a,** 4 µS, and **b,** 40 µS. **c,** Accuracy of inference measured experimental and calculated via a model for two distortion levels (10% and 50%). **d,** Experimentally measured inference accuracy with and without encryption for 10% distortion. The task was a reconstruction of Braille representations of the names of nine famous computer scientists: Hamilton, Lovelace, Hopper, Turing, Antonelli, Shannon, Ritchie, McCarthy and Neuman. The task, training, and inference procedure are detailed elsewhere[12].

Supplementary Note 7. Homomorphic encryption and federated learning.

Homomorphic encryption (HE) is a form of encryption that allows one to perform computations directly on encrypted data[13,14]. The two types of HE are addition and multiplication. Our encryption method facilitates a simple additively HE, where encrypted XOR operation results in an output identical to what would have been generated if the operation was performed on the raw data (Supplementary Fig. 34a). Performing multiplicatively HE is more challenging and requires a complex operation beyond simple Boolean logic functions. One way to do this is the matrix multiplication using the memristive crossbar[15–18], shown in the encrypted inference part.

Based on HE using our proposed encryption process, it can be implemented for federated learning (FL). FL is a decentralized machine learning technology that allows distributed devices to collaboratively train a learning model without data exchange, thus achieving privacy[19–22]. It is designed to train a model combining parties' data by sharing model parameters rather than the actual local data, preserving the parties' data. It is essential in any field that processes privacy-sensitive data. For instance, after experiencing the recent pandemic of COVID-19, training a healthcare model has become important for both the hospital and healthcare industry to provide better medical solutions[21]. In this case, several hospitals can use FL to train the model cooperatively without revealing patient privacy. There are several other applications of federated learning, such as smartphones, organizations, and the Internet of Things[20]. However, the model parameters can be reverse-engineered to discover the raw data, leading to severe privacy threats. Here, a HE-based FL system is proposed by adopting the encryption scheme. Adding HE in FL can efficiently guarantee data privacy and security. The proof of concept for the HE-based FL system is shown in Supplementary Fig. 34b. Initially, a global model defined by a server is distributed to parties holding local models. The parties share a private key that is used to encrypt or decrypt their data and also share with the server a public key. After training the local models, the updated parameters are encrypted with the private key, returning them to

the server. The server updates the global model parameters using the locally trained model parameters, and the public key is used to encrypt the aggregated data. Then, the new encrypted model is delivered back to the parties and can be decrypted with the public key for another local training. This process is repeated until convergence is achieved. Implementing the simple additively HE method, even though the result of the additively HE is decrypted data, the raw data are still unknown, thereby maintaining privacy. Since the PUF key is random and unpredictable, it is resistant to any inversion attack. The aggregated data can be encrypted with the public key and sent back to the local models, so the entire training can be privacy-preserving. This privacy-preserving system using memristors may provide a hardware solution for the new machine learning technology.

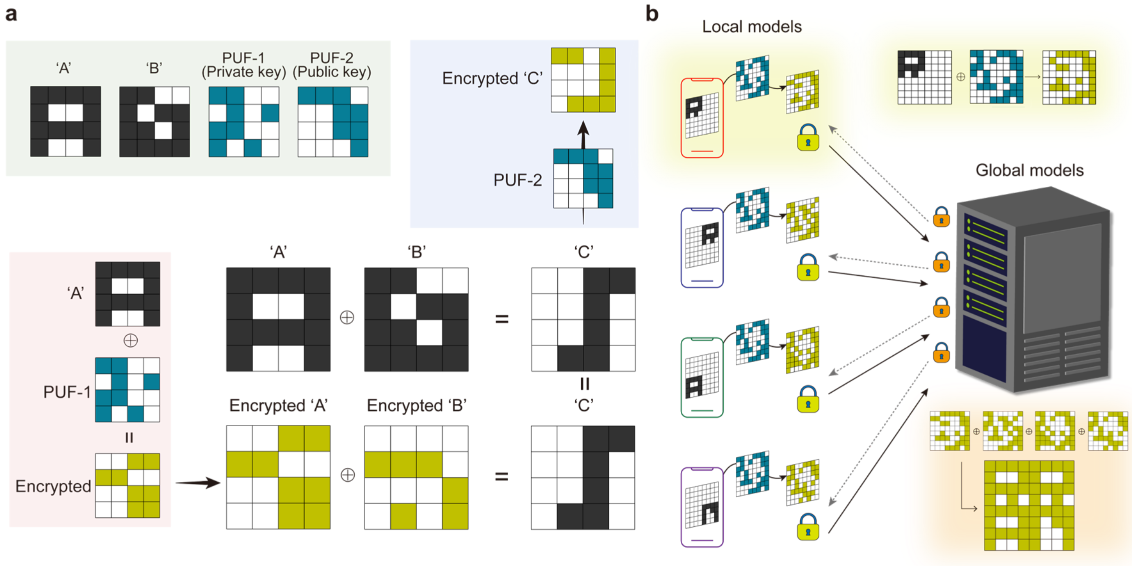

Supplementary Fig. 34 | a, Homomorphic encryption based on XOR. 'A' and 'B'-shaped data (local models) are encrypted using a private key (PUF-1). Addition involves XOR operation, and the additively homomorphic encryption yields an output identical to what would have been produced through the execution of the same operation on the unencrypted 'A' and 'B'. If it was implemented for federated learning, the aggregated data can be encrypted with a public key (PUF-2), and sent back to the local models. **b,** Proof of concept for homomorphic-encryption-based federated learning system. A simple form of homomorphic encryption is shown.

Supplementary Note 8. Energy and latency comparisons of memristor-based encrypted inference with traditional computing systems.

8.1. Energy/latency for key generation.

The performance of CMOS-based digital computers for random number generation (RNG) is estimated from a recent work by Intel[23]. They demonstrated various operation modes, each being optimized for a different quantity. The standard mode produced 162.5 Mb/s, with 1.5 mW of power consumption, yielding an energy efficiency of 9 pJ/bit. Mode (vi) was optimized for energy efficiency, with a peak encrypted bit throughput of 323 Gbps/W (or 323 Gb/J), with an energy efficiency of 3 pJ/bit. Thus, in mode (i), 144 pJ is consumed during 94 ns to generate 16 random binary numbers.

The energy and latency calculations (Supplementary Fig. 35) of PUF-1 for the memristor were performed based on our hardware setup described in Supplementary Fig. 30.

8.2. Energy/latency for encryption/decryption by XOR.

Nguyen et al. performed computer architectural studies for the energy benchmarking of CMOS-based XOR gate operation[24,25]. The 4-bit carry-lookahead adder (CLA) in Supplementary Fig. 36a constitutes the basic building block of the simulated CMOS-based 32-bit parallel adders with 8 XOR gates, 14 AND gates, and 4 OR gates (including a two-level carry-lookahead box with dotted line). Eight rippled-4-bit CLAs form a 32-bit adder (Supplementary Fig. 36b), of which 32 replications and 8 kB L1 cache memory (Supplementary Fig. 36c), form a cluster to perform 32 additions simultaneously. One cluster with 32 cores consumes 1.2 μJ from the computer architecture simulation[25], and this value was adopted to calculate the energy consumption of a logic gate from CMOS transistors assuming similar energy consumptions (6 transistors per gate regardless of XOR, AND, and OR). Therefore, the number of logic gates per cluster is 6656 (= 32 adders × 8 CLAs × 26 gates), and the energy consumption per logic gate is 0.18 nJ. The number of total XOR operations used in the present

demonstration ('A'-shaped data) is 16, so the total energy consumption from the encryption/decryption is 2.88 nJ. The latency for one XOR operation is 9 ps (0.14 ns for 16 XOR operations). For XOR encryption/decryption using the memristive system, the energy and latency calculations of encrypting 'A'-shaped data were performed based on the hardware setup described in Supplementary Fig. 30 (Supplementary Fig. 35). Table 2 summarizes the performance associated with the XOR encryption.

Element	Energy (pJ)	Latency (ns)
Memristor write	0.0165	1.000
DAC	0.0400	0.150
ADC	0.0150	0.150
TIA / Amplifier	1.0000	0.300
MUX	0.2740	0.004

Random Number Generation *Single operation*

Element	MUX	DEMUX	DAC	TIA	MEM	total
Energy (pJ)	0.5480	0.5480	0.0400	0.1250	0.0165	2.1525
Latency (ns)	0.008	0.008	0.150	0.300	1.000	1.466

Step total energy (pJ) = 34.440
Step total latency(ns) = 23.456

XOR logic operation (1/3) *Single operation*

Element	MUX#	DEMUX	DAC	TIA	MEM	total
Energy (pJ)	0.8220	0.5480	0.0400	1.0000	0.0165	2.15250
Latency (ns)	0.012	0.008	0.150	0.300	1.000	1.466

Step total energy (pJ) = 34.988
Step total latency(ns) = 23.464

#Including write mode switch

XOR logic operation (2/3) *Single operation*

Element	MUX	DEMUX	DAC	TIA	MEM	01, 10 op.	11 op.	00 op.
Energy (pJ)	0.5480	0.5480	0.0400	1.0000	0.0165	2.1525	2.1760	1.0960
Latency (ns)	0.008	0.008	0.150	0.300	1.000	1.466	0.616	0.016

Step total energy (pJ) = 32.468
Step total latency(ns) = 15.456

XOR logic operation (3/3) *Single operation*

Element	MUX	DEMUX	DAC	TIA	MEM	01, 10 op.	11 op.	00 op.
Energy (pJ)	0.5480	0.5480	0.04000	1.0000	0.01650	2.1525	2.1760	1.0960
Latency (ns)	0.008	0.008	0.150	0.300	1.000	1.466	0.616	0.016

Step total energy (pJ) = 32.468
Step total latency(ns) = 15.456

Supplementary Fig. 35 | Energy and latency of memristor-based key generation and XOR encryption for encrypting A-shaped data with PUF-1, as shown in Supplementary Fig. 29. The performance quantities are projected to 16 nm scaled sizes using standard foundry scaling rules, which is detailed elsewhere[24,25].

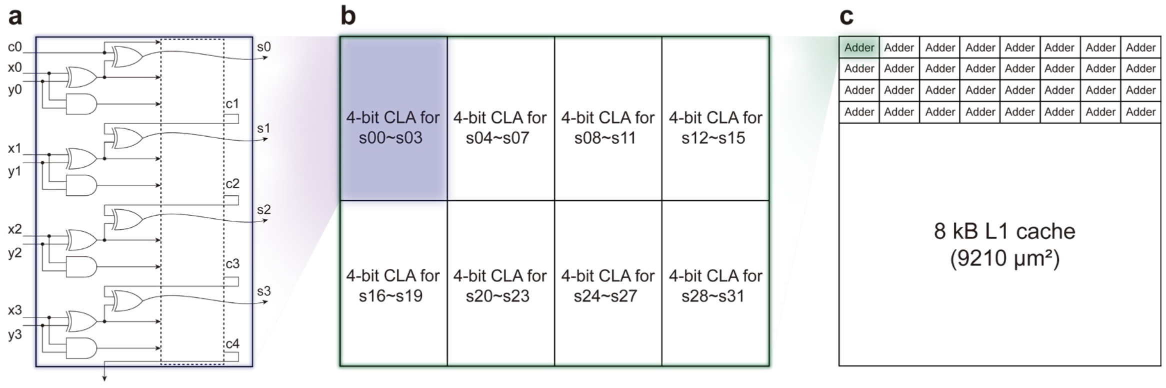

Supplementary Fig. 36 | Energy benchmark of an XOR gate from CMOS technology with full hierarchy including adders, cache memory, and dynamic random-access memory. a, 4-bit carry lookahead adder, 8 replications of which forms a 32-bit adder. **b,** 32-bit adder, 32 replications (core) of which form a cluster of multicore 32-bit parallel adder. **c,** A cluster that is capable of performing 32 additions simultaneously by using 32 adders and 8kB L1 cache memory.

Supplementary Table 4. Sputtering power for various Cu_xTe_{1-x} devices.

Composition	Cu power	Te power
$Cu_{0.1}$ $Te_{0.9}$	10 W	120 W
$Cu_{0.2}$ $Te_{0.8}$	15 W	80 W
$Cu_{0.3}$ $Te_{0.7}$	20 W	55 W
$Cu_{0.4}$ $Te_{0.6}$	25 W	45 W
$Cu_{0.6}$ $Te_{0.4}$	50 W	40 W
$Cu_{0.77}$ $Te_{0.23}$	75 W	40 W

Supplementary References

1. Woo, K. S. *et al.* A High-Speed True Random Number Generator Based on a Cu_xTe_{1-x} Diffusive Memristor. *Adv. Intell. Syst.* **3**, 2100062 (2021).
2. Wang, W. *et al.* Surface diffusion-limited lifetime of silver and copper nanofilaments in resistive switching devices. *Nat. Commun.* **10**, (2019).
3. Mullins, W. W. Theory of Thermal Grooving. *J. Appl. Phys.* **28**, 333–339 (1957).
4. Kim, Y. S., Son, M. W. & Kim, K. M. Memristive Stateful Logic for Edge Boolean Computers. *Adv. Intell. Syst.* **3**, 2000278 (2021).
5. Huang, P. *et al.* Reconfigurable Nonvolatile Logic Operations in Resistance Switching Crossbar Array for Large-Scale Circuits. *Adv. Mater.* **28**, 9758–9764 (2016).
6. Adam, G. C., Hoskins, B. D., Prezioso, M. & Strukov, D. B. Optimized stateful material implication logic for three-dimensional data manipulation. *Nano Res.* **9**, 3914–3923 (2016).
7. Cheng, L. *et al.* Reprogrammable logic in memristive crossbar for in-memory computing. *J. Phys. D: Appl. Phys.* **50**, (2017).
8. Kim, K. M. *et al.* Single-Cell Stateful Logic Using a Dual-Bit Memristor. *Phys. Status Solidi RRL* **13**, (2019).
9. Xu, L. *et al.* Memristor-Based Efficient In-Memory Logic for Cryptologic and Arithmetic Applications. *Adv. Mater. Technol.* **4**, (2019).
10. Xu, N. *et al.* A Stateful Logic Family Based on a New Logic Primitive Circuit Composed of Two Antiparallel Bipolar Memristors. *Adv. Intell. Syst.* **2**, 1900082 (2020).
11. Cai, F. *et al.* A Fully Integrated System-on-Chip Design with Scalable Resistive Random-Access Memory Tile Design for Analog in-Memory Computing. *Adv. Intell. Syst.* **4**, 2200014 (2022).
12. Yi, S. in, Kendall, J. D., Williams, R. S. & Kumar, S. Activity-difference training of deep neural networks using memristor crossbars. *Nat. Electron.* **6**, 45–51 (2023).
13. Cheon, J. H., Kim, A., Kim, M. & Song, Y. Homomorphic Encryption for Arithmetic of Approximate Numbers. in *23rd International Conference on the Theory and Applications of Cryptology and Information Security Hong Kong* 409–437 (2017).
14. Ibarrondo, A. & Viand, A. Pyfhel: PYthon for Homomorphic Encryption Libraries. in *WAHC 2021 - Proceedings of the 9th Workshop on Encrypted Computing and Applied Homomorphic Cryptography, co-located with CCS 2021* 11–16 (Association for Computing Machinery, Inc, 2021). doi:10.1145/3474366.3486923.
15. Li, C. *et al.* Analogue signal and image processing with large memristor crossbars. *Nat. Electron.* **1**, 52–59 (2018).
16. Hu, M. *et al.* Memristor-Based Analog Computation and Neural Network Classification with a Dot Product Engine. *Adv. Mater.* **30**, (2018).
17. Li, C. *et al.* Efficient and self-adaptive in-situ learning in multilayer memristor neural networks. *Nat. Commun.* **9**, (2018).
18. Cai, F. *et al.* A fully integrated reprogrammable memristor–CMOS system for efficient multiply–accumulate operations. *Nat. Electron.* **2**, 290–299 (2019).
19. Brendan McMahan Eider Moore Daniel Ramage Seth Hampson Blaise AgüeraAg, H. & Arcas, A. *Communication-Efficient Learning of Deep Networks from Decentralized Data*. (2017).
20. Li, T., Sahu, A. K., Talwalkar, A. & Smith, V. Federated Learning: Challenges, Methods, and Future Directions. *IEEE Signal Process. Mag.* **37**, 50–60 (2020).
21. Rieke, N. *et al.* The future of digital health with federated learning. *NPJ Digit. Med.* **3**, (2020).

22. Li, L., Fan, Y., Tse, M. & Lin, K. Y. A review of applications in federated learning. *Comput. Ind. Eng.* **149**, (2020).
23. Mathew, S. K. *et al.* µRNG: A 300-950 mV, 323 Gbps/W All-Digital Full-Entropy True Random Number Generator in 14 nm FinFET CMOS. *IEEE J. Solid-State Circuits* **51**, 1695–1704 (2016).
24. Hoang Anh Du Nguyen *et al.* Computation-In-Memory Based Parallel Adder. in *IEEE/ACM International Symposium on Nanoscale Architectures (NANOARCH)* 198 (2015).
25. Nguyen, H. A. Du *et al.* On the Implementation of Computation-in-Memory Parallel Adder. *IEEE Trans. Very Large Scale Integr. VLSI Syst.* **25**, 2206–2219 (2017).
